# A Low‐Temperature Synthetic Route Toward a High‐Entropy 2D Hexernary Transition Metal Dichalcogenide for Hydrogen Evolution Electrocatalysis

**DOI:** 10.1002/advs.202204488

**Published:** 2023-03-23

**Authors:** Jie Qu, Amr Elgendy, Rongsheng Cai, Mark A. Buckingham, Athanasios A. Papaderakis, Hugo de Latour, Kerry Hazeldine, George F. S. Whitehead, Firoz Alam, Charles T. Smith, David J. Binks, Alex Walton, Jonathan M. Skelton, Robert A. W. Dryfe, Sarah J. Haigh, David J. Lewis

**Affiliations:** ^1^ Department of Materials The University of Manchester Oxford Road Manchester M13 9PL UK; ^2^ Department of Chemistry and Sir Henry Royce Institute The University of Manchester Oxford Road Manchester M13 9PL UK; ^3^ Department of Materials National Graphene Institute and Sir Henry Royce Institute The University of Manchester Oxford Road Manchester M13 9PL UK; ^4^ Department of Chemistry and the Photon Science Institute The University of Manchester Oxford Road Manchester M13 9PL UK; ^5^ Department of Chemistry The University of Manchester Oxford Road Manchester M13 9PL UK; ^6^ Department of Physics and Astronomy and the Photon Science Institute The University of Manchester Oxford Road Manchester M13 9PL UK

**Keywords:** 2D material, high‐entropy disulfide, hydrogen evolution reaction electrocatalysis, single source precursor

## Abstract

High‐entropy (HE) metal chalcogenides are a class of materials that have great potential in applications such as thermoelectrics and electrocatalysis. Layered 2D transition‐metal dichalcogenides (TMDCs) are a sub‐class of high entropy metal chalcogenides that have received little attention to date as their preparation currently involves complicated, energy‐intensive, or hazardous synthetic steps. To address this, a low‐temperature (500 °C) and rapid (1 h) single source precursor approach is successfully adopted to synthesize the hexernary high‐entropy metal disulfide (MoWReMnCr)S_2_. (MoWReMnCr)S_2_ powders are characterized by powder X‐ray diffraction (pXRD) and Raman spectroscopy, which confirmed that the material is comprised predominantly of a hexagonal phase. The surface oxidation states and elemental compositions are studied by X‐ray photoelectron spectroscopy (XPS) whilst the bulk morphology and elemental stoichiometry with spatial distribution is determined by scanning electron microscopy (SEM) with elemental mapping information acquired from energy‐dispersive X‐ray (EDX) spectroscopy. The bulk, layered material is subsequently exfoliated to ultra‐thin, several‐layer 2D nanosheets by liquid‐phase exfoliation (LPE). The resulting few‐layer HE (MoWReMnCr)S_2_ nanosheets are found to contain a homogeneous elemental distribution of metals at the nanoscale by high angle annular dark field‐scanning transmission electron microscopy (HAADF‐STEM) with EDX mapping. Finally, (MoWReMnCr)S_2_ is demonstrated as a hydrogen evolution electrocatalyst and compared to 2*H*‐MoS_2_ synthesized using the molecular precursor approach. (MoWReMnCr)S_2_ with 20% w/w of high‐conductivity carbon black displays a low overpotential of 229 mV in 0.5 M  H_2_SO_4_ to reach a current density of 10 mA cm^−2^, which is much lower than the overpotential of 362 mV for MoS_2_. From density functional theory calculations, it is hypothesised that the enhanced catalytic activity is due to activation of the basal plane upon incorporation of other elements into the 2*H*‐MoS_2_ structure, in particular, the first row TMs Cr and Mn.

## Graphical abstract

A novel high entropy sulfide (MoWReMnCr)S_2_ is synthesized by facile thermal decomposition of a cocktail of five individual molecular precursors at a relatively low temperature of 500 °C for 1 h. The performance of the exfoliated 2D nanosheets derived from these high entropy layered materials are measured for electrocatalytic activity in the hydrogen evolution reaction (HER) and the results compared to the widely‐studied electrocatalyst  2*H*‐MoS_2_.

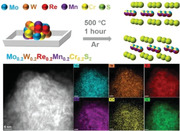

## 1 Introduction

Since the successful synthesis of single atom‐thick graphene from graphite in 2004,^[^ ^1^ ^]^ 2D materials have become an area of substantial research interest.^[^ ^2^ ^]^ This family of materials has subsequently expanded beyond graphene to include metal oxides, main‐group metal chalcogenides, and transition‐metal dichalcogenides (TMDCs).^[^ ^3^ ^]^ Inorganic 2D nanomaterials have been used for a wide variety of applications including electronics,^[^ ^4^ ^]^ energy conversion,^[^ ^3a,d^ ^]^ catalysis,^[^ ^3^, ^5^ ^]^ magnetic materials,^[^ ^6^ ^]^ and sensing.^[^ ^7^ ^]^ 2D MoS_2_ is an inorganic grapheneanalogue that exists in 1*T* (trigonal), 2*H* (hexagonal), and 3*R* (rhombohedral) polymorphs which are distinct from each other by differing coordination between Mo and S atoms in the unit cell.^[^ ^8^ ^]^ 1*T*‐MoS_2_ is metallic and is known to be an excellent electrocatalyst for the hydrogen evolution reaction (HER) but is not thermodynamically stable . 2*H*‐MoS_2_ is a semiconductor, but only the edge and defect sites contribute appreciably to its electrocatalytic activity while the majority basal‐plane sites are considered to be relatively inactive.^[^ ^9^ ^]^ Ternary MoS_2_‐based materials for hydrogen‐evolution electrocatalysis such as Mo_1−_ *_x_* W*_x_* S_2_,^[^ ^10^ ^]^ Mo_1−_ *_x_* Re*_x_* S_2_,^[^ ^11^ ^]^ and MoS_2−_ *_x_* Se*_x_* ^[^ ^12^ ^]^ have been studied and have demonstrated improved electrocatalytic performance compared to pure 2*H*‐MoS_2_, with the incorporation of multiple alloying elements shown to activate the basal sites for H^+^ adsorption, enhancing the electrocatalytic activity significantly.^[^ ^13^ ^]^

High‐entropy materials (HEMs) are materials that contain six or more constituent elements and when only one sub‐lattice is disordered, such as in MS_2_ with five or more metals distributed over the M sites, the entropic stabilisation increased compared to the binary materials when each element is present at 20 mol%.^[^ ^14^ ^]^ HEMs were first proposed in the context of multicomponent / high entropy alloys (HEAs),^[^ ^15^ ^]^ and this unique family of materials was subsequently expanded to include HE metal oxides,^[^ ^16^ ^]^ borides,^[^ ^17^ ^]^ carbides,^[^ ^18^ ^]^ nitrides,^[^ ^19^ ^]^ silicides,^[^ ^20^ ^]^ phosphides/phosphates,^[^ ^21^ ^]^ fluorides,^[^ ^22^ ^]^ and chalcogenides.^[^ ^14^, ^23^ ^]^ These materials are of interest as the high configurational entropy of mixing^[^ ^24^ ^]^ typically results in enhanced physical properties (e.g. increased melting point, hardness) whilst the combinatorial complexity of the material results in the so‐called cocktail effect , which leads to low thermal transport,^[^ ^25^ ^]^ high electrical transport,^[^ ^25^, ^26^ ^]^ and enhanced catalytic activity.^[^ ^27^ ^]^ Recently, the entropic stabilization of 2D materials has been investigated in an attempt to access new families of synthetic nanomaterials with emergent properties in the 2D limit.^[^ ^27^, ^28^ ^]^ If this concept can be extended to TMDCs , it might confer the beneficial properties associated with HEMs on this class of materials. To date, there has only been one report of a HE 2D MoS_2_‐based high entropy material—with 8–16 atomic layers, which has been demonstrated for carbon dioxide electrocatalysis.^[^ ^27a^ ^]^ However, the preparation of this material requires highly toxic HF etching and annealing of the reaction vessel at 1000 °C, followed by a 120 h elemental annealing procedure on the component powders to generate the HE material. Even after this tortuous homogenization procedure, the materials show evidence of spatial separation of elements at the nanoscale from the energy‐dispersive X‐ray spectroscopy (EDX) spectroscopic imaging presented, and no diffraction data is presented that may be able to quantify the effect of this on the structure. This approach may also be incompatible with the production of materials with emergent 2D electronic effects as it has previously been shown that bulk‐like behavior is observed in MoS_2_ with as few as eight layers.^[^ ^29^ ^]^ Thus, an effective and scalable synthetic route toward few‐layer TMDC‐based HEMs is required to address these problems.

In this work, we present a new approach to the synthesis of a MoS_2_‐based HEM through the thermal decomposition of a cocktail of five molecular precursors. This precursor approach has a number of advantages including mixing of precursors at the atomic scale to maximize the disorder in the solid‐state products, relatively short processing times, and relatively low preparation temperatures (<1000 °C).^[^ ^30^ ^]^ The approach is a significant improvement to both the time and energy required to synthesize HE chalcogenides (Table S1, Supporting Information). Powder X‐ray diffraction (pXRD) and Raman spectroscopy demonstrate that (MoWReMnCr)S_2_ can be structurally stabilized in the 2*H* phase. Subsequent liquid‐phase exfoliation (LPE) of the bulk HE metal disulfide yields the 2D HE material with 3–7 atomic layers, which we confirm using high angle annular dark field (HAADF)‐scanning transmission electron microscopy (STEM) analysis. Last, the entropically‐stabilized 2D (MoWReMnCr)S_2_ nanosheets are investigated as electrocatalysts for HER, and found to deliver significantly enhanced electrocatalytic activity compared with parent 2*H*‐MoS_2_, prepared through the same route. This new approach, which blends elements from bottom–up (tandem decomposition of single‐source precursors) and top–down (LPE to few‐layered 2D materials) processing in synergy, unlocks an effective and scalable synthetic route toward 2D MoS_2_‐based HEMs with the potential to be extended to the synthesis of other HE 2D TMDCs.

## 2 Results and Discussion

**Figure 1:**
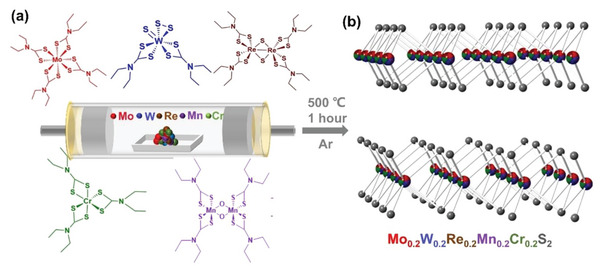
a) Schematic of the preparation of the high‐entropy transition metal disulfide, where five individual precursors are decomposed in tandem to form the HE material. b) Crystal structure of the HE transition metal disulfide showing variable occupancy of the metal sites within the 2*H*‐MoS_2_ structure (indicated by the multi‐colored spheres).

**Figure 2:**
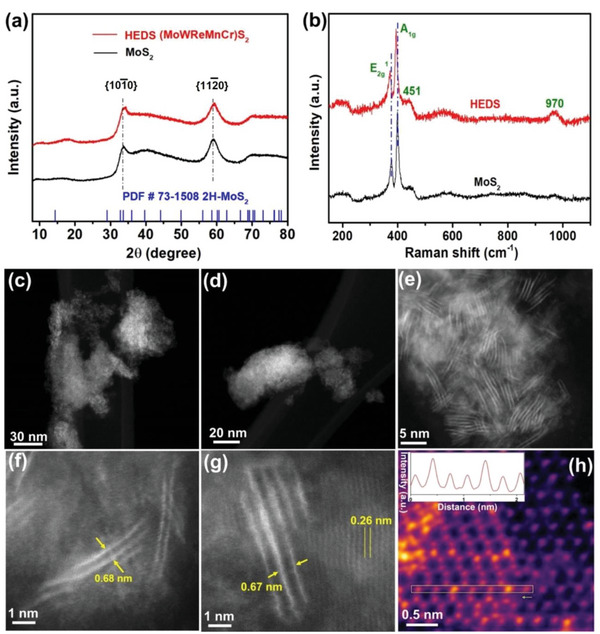
Structural characterization of bulk (MoWReMnCr)S_2_ and the exfoliated 2D nanosheets. a) pXRD patterns of the bulk (MoWReMnCr)S_2_ (designated in figure as 'high entropy disulfide' ‐ HEDS) and pure MoS_2_ prepared using the same method. The reference peak positions expected for 2*H*‐MoS_2_ are also shown for comparison. b) Raman spectra of bulk (MoWReMnCr)S_2_ and MoS_2_. c–h) STEM‐HAADF images of exfoliated 2D nanosheets of (MoWReMnCr)S_2_ at various magnifications showing varying numbers of randomly distributed layers (c,d), the interlayer distances between atomic planes (f,g), and the plan view atomic structure (h), falsely colored to highlight variations in the intensity of neighboring atomic columns. The inset in (h) shows the variation in pixel intensity along the area marked in the green box.

Synthesis of bulk (MoWReMnCr)S_2_ was performed by the tandem thermal decomposition of five individual metal dithiocarbamate precursors, the chemical structures of which are shown in **Figure** **1**a; characterization is reported in Figures S1–S6, Supporting Information. This approach has been previously used to synthesize a range of ternary and quaternary metal sulfides,^[^ ^31^ ^]^ but to date has only been used once to produce a high‐entropy material of multi‐lanthanide oxysulfides^[^ ^30^ ^]^ and at high temperatures (≈900 °C). We therefore attempted to adapt this approach to the preparation of 2D HE metal dichalcogenides. Powders of the five individual precursors were combined in equal ratio and thermally decomposed at a relatively low temperature (500 °C) for 1 h under an argon atmosphere, as shown schematically in Figure 1a. The resultant bulk particulate material was analyzed using powder X‐ray diffraction (pXRD, **Figure** **2**a). Two peaks were observed at 2*θ* = 33° and 58°, that can be attributed to the {$10\bar{1}0$} and {$11\bar{2}0$} planes of a layered solid with a similar structure to 2*H*‐MoS_2_^[^ ^32^ ^]^ (Figure 1b). Indeed, the data for the (MoWReMnCr)S_2_ is consistent with X‐ray data previously reported by Lewis and co‐workers^[^ ^8c^ ^]^ for 2*H*‐MoS_2_ synthesized by direct thermolysis of molybdenum dithiocarbamates.

We also noted a distinct lack of any (000*l*) reflections observed in these patterns, which is consistent with the thinning of the materials to atomic dimensions along the *c* direction,^[^ ^8^, ^33^ ^]^ and the extensive Scherrer broadening of the peaks further suggests that the materials were also confined to nanoscale dimensions in the *a‐b* directions. We confirmed the elemental composition and spatial distribution at the microscale using scanning electron microscopy (SEM) with energy‐dispersive X‐ray (EDX) spectroscopy (Figure S7, Supporting Information). All metals appeared to be evenly distributed, with the determined elemental compositions reported in Table S2, Supporting Information. The material produced was found to contain significant portions of all five metals but was slightly deficient in Re, Mn, and Cr and rich in Mo and W compared to the nominally equimolar ratios in the starting precursor mixture. To ascertain the oxidation states of the elements within the material, XPS analysis was performed. As shown in Figures S8 and S9, Supporting Information, the characteristic signals for Mo, W, Re, Mn, Cr, and S could be clearly identified (survey XPS spectra are also shown in Figure S10, Supporting Information). The parent MoS_2_ was found to have 71.6% sulfur content (inset table in Figure S10a, Supporting Information), which decreased to 60.2% in the high entropy material (Figure S10b, Supporting Information). A full discussion of the oxidation states of all the elements present and fitting of the XPS data can be found in the Supporting Information.

The Raman spectra of both the high entropy material and MoS_2_ are shown in Figure 2b. The in‐plane E_2g_^1^ vibrational mode (≈378 cm^−1^) and out‐of‐plane mode A_1g_ mode (≈400 cm^−1^)^[^ ^34^ ^]^ can be clearly detected in both samples. However, the positions of these two characteristic peaks are slightly lower in the high entropy material compared to the parent MoS_2_, which suggests a softening of the Mo—S phonon mode in the basal planes, which has previously been ascribed to reduced Mo—S bonding.^[^ ^35^ ^]^ The Raman spectroscopy again confirms the successful synthesis of a HE 2*H*‐MoS_2_ phase, in agreement with the pXRD data.^[^ ^36^ ^]^ The characteristic peaks expected from bulk MoO_3_^[^ ^37^ ^]^ were not found in the Raman analysis, indicating that the oxides observed in the XPS analysis (Figure S9, Supporting Information) are limited to the surface and likely introduced by surface oxidation. The synthesis of a phase‐pure 2*H* MoS_2_ lattice containing high concentrations of Cr and Mn is a significant observation. Mn is difficult to dope into MoS_2_ in high quantities, as it tends to form 3D MnS and destroys the layer structure;^[^ ^38^ ^]^ yet, here we have successfully introduced high concentrations of both Mn (5.0 mol%) and Cr (4.7 mol%) into 2*H* MoS_2_. We speculate that this is made possible through entropic stabilization from the high configurational entropy of the multicomponent system.^[^ ^39^ ^]^

The similarity of bonding in the layered HE material to MoS_2_ suggested that exfoliation to the 2D limit should be achievable. Liquid‐phase exfoliation (LPE) is a scalable method of producing 2D (but predominantly few‐layer) materials from their bulk counterparts requiring only simple apparatus.^[^ ^40^ ^]^ To test this hypothesis, we therefore performed LPE on the bulk HE material in N‐methyl pyrrolidine for 24 h. The resulting few‐layer material was analyzed using high angle annular dark field‐scanning transmission electron microscopy (HAADF–STEM) (Figure 2c–h). The images reveal agglomerates of few‐layer HE metal dichalcogenide nanosheets were produced with diameters of 10–50 nm (Figure 2c,d). High‐resolution (HR) HAADF–STEM images show that the exfoliated nanoflakes can be seen with two orientations on the support, i) with the basal plane aligned vertically parallel with the electron beam and ii) lying horizontally perpendicular to the electron beam (Figure 2e–g). From flakes with the former orientation, the interlayer distance between atomic planes in the HE metal dichalcogenide sheets was determined to be ≈0.7 nm (Figure 2f,g), which is consistent with the material having the parent 2*H*‐MoS_2_ structure.^[^ ^41^ ^]^ From flakes with the horizontal orientation with respect to the electron beam, the hexagonal atomic arrangement in 2D HE metal disulfide basal plane (Figure 2h; Figure S11, Supporting Information) was observed, with the *d* spacing of 0.26 nm in Figure 2g indexed to the {$10\bar{1}0$} plane which is also consistent with the 2*H*‐MoS_2_.^[^ ^8^, ^42^ ^]^ Analyzing the exfoliated material at the atomic scale by Z‐contrast STEM showed variations in the atomic column intensities, consistent with atomic alloying and local cation variations as heavier elements (Re and W) give higher HAADF intensity (Figure 2h inset).

**Figure 3:**
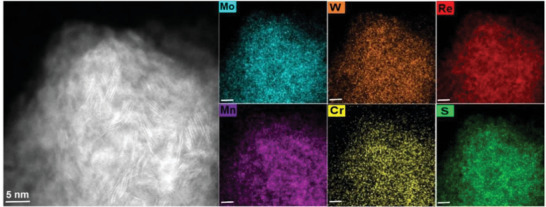
Elemental distribution in agglomerated 2D nanosheets of (MoWReMnCr)S_2_: HAADF‐STEM image (left) and corresponding EDX elemental maps of the Mo L*α*, W L*α*, Re L*α*, Mn K*α*, Cr K*α*, and S K*α* emission. The scale bars in each image are 5 nm.

The exfoliated 2D material was further analyzed by atomic force microscopy (AFM). Figure S13, Supporting Information, shows the topography of the exfoliated 2D materials deposited on a quartz substrate at different length scales. Taking a height profile of one of the particles, a height of ≈3 nm was determined (Figure S6c, Supporting Information). This demonstrates that thin material can be produced through a relatively simple synthesis and exfoliation technique. Together with the few‐layer thicknesses seen in the HAADF‐STEM images in Figure 2e–g, this suggests that with further purification (e.g., by liquid cascade centrifugation^[^ ^43^ ^]^), it should be possible to separate few‐layer components out and further purify the monolayer material, but this is beyond the scope of the current study in which we sought only to establish whether exfoliation is indeed possible regardless of the extent of size dispersion. STEM‐EDX was used to investigate the elemental distribution within the exfoliated material (**Figure** **3**; Figure S14, Supporting Information), showing mainly homogenous distribution of all elements at the nanoscale, but with some minimal localized clustering of metals, consistent with the literature on HE materials.^[^ ^24^, ^27^, ^44^ ^]^ The quantified elemental compositions obtained from the summed STEM‐EDX spectra is shown in Tables S3 and S4, Supporting Information, and are consistent with the composition determined by SEM‐EDX at the microscale.

**Figure 4:**
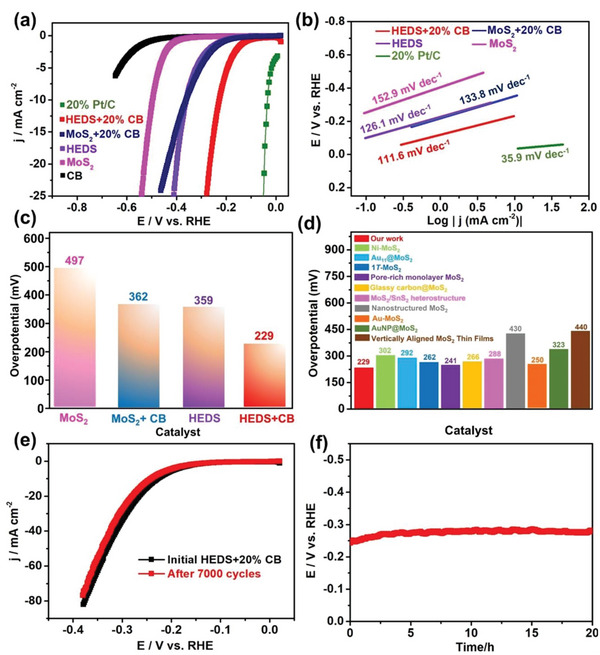
Electrochemical tests of the as‐prepared (MoWReMnCr)S_2_ nanosheets (designated in figure as 'high entropy disulfide' ‐ HEDS) with and without conductive carbon (CB) and 20% Pt/C in 0.5 m H_2_SO_4_. a) Linear sweep voltammograms (LSVs) within the HER potential range at a scan rate of 5 mV s^−1^. b) Tafel plots generated from the LSV curves. c) *η*_10_ values of the as‐prepared materials. d) Comparison of the *η*_10_ values with previously‐reported MoS_2_‐based catalysts. e) LSV curves of a HED@20% CB electrode before and after continuous cycling between −0.2 and −0.6 V versus Ag/AgCl for 7000 cycles at a scan rate of 200 mV s^−1^. f) Durability test for 20 h at 10 mA cm^−2^ current density recorded using the chronopotentiometry technique. All areas are geometric surface areas.

The catalytic performance of the as‐prepared (MoWReMnCr)S_2_ nanosheets toward the hydrogen‐evolution reaction (HER) was evaluated and compared to the parent MoS_2_ material synthesized using the same method and to the benchmark 20% Pt/C. HER activity was investigated by means of iR‐compensated polarization curves recorded using linear sweep voltammetry (LSV). As shown in **Figure** **4**a,c, the (MoWReMnCr)S_2_ nanosheets exhibit an onset potential (defined as the potential required to reach 1 mA cm^−2^) of −250 mV, which is significantly lower than that of the MoS_2_ (≈ −400 mV). The overpotential required to reach 10 mA cm^−2^ (*η*_10_) was determined to be 359 ± 6 mV for the (MoWReMnCr)S2 electrode, which is 28% lower than the overpotential of 497 ± 9 mV, recorded for MoS_2_.

In contrast to metallic 1*T*‐MoS_2_, pristine 2*H*‐MoS_2_ is a semiconductor, and the edge and defect sites are responsible for its catalytic activity while the basal‐plane is considered inactive. In an attempt to improve the conductivity of the prepared electrode films, we incorporated commercially‐available highly‐conductive carbon (CB) into the pristine material at a 20% w/w ratio by mixing the two materials in the solid state. The conductive carbon not only provides facile electron transport but also reduces the self‐stacking of the (MoWReMnCr)S_2_ and 2*H*‐MoS_2_ nanosheets; and thus, increases the number of active sites.^[^ ^45^ ^]^ We also noted that the addition of 20% w/w CB facilitates the dispersion of active materials, resulting in a homogenous ink. Mixing with CB has been previously used with several Mo and S‐based semiconducting electrocatalysts, including MoS_2_,^[^ ^46^ ^]^ amorphous MoS_x_,^[^ ^45^ ^]^ and more recently Chevrel phase Mo_6_S_8_.^[^ ^47^ ^]^ After mixing the pristine (MoWReMnCr)S_2_ with CB, we obtained a low onset potential of −127 mV (Figure 4a,c), which was significantly lower than the comparable MoS_2_@20%CB potential of −219 mV. Pure CB shows negligible catalytic activity toward HER, demonstrating that the improved catalytic performance of the (MoWReMnCr)S_2_ material on mixing with CB can be attributed to the intrinsic physicochemical properties of the latter and the increased number of catalytic sites. Among all the samples studied, the (MoWReMnCr)S_2_@20%CB electrode shows the best HER activity with an *η*_10_ of −229 ± 5 mV, which is 130 mV lower than that of the pristine (MoWReMnCr)S_2_. This overpotential value is significantly lower than those reported for nanostructured 2*H*‐MoS_2_^[^ ^48^ ^]^ and even for some metallic 1*T*‐MoS_2_ materials.^[^ ^49^ ^]^ Furthermore, it is also noteworthy that the prepared (MoWReMnCr)S_2_@20%CB outperforms previously‐reported MoS_2_ materials with different structures in terms of HER performance (Figure 4d; Table S6, Supporting Information). The high electrocatalytic performance of the (MoWReMnCr)S_2_ might therefore be attributed to the cocktail effect.

To establish the origin of the high electrocatalytic performance toward the HER in (MoWReMnCr)S_2_, density‐functional theory (DFT) calculations were performed on a series of slab models of the basal plane MoS_2_ (001) surface with up to four of the surface Mo atoms substituted by Cr, Mn, W, or Re. We investigated bare MoS_2_ plus a series of 19 substituted models comprising single substitutions with each of the four elements (four models), double substitutions with two of the same (four models) or two different elements (six models), triple substitutions with three different elements (four models), and a quadruple substitution with one each of the four elements (one model). For each model, we determined the lowest‐energy arrangement of the substituting atoms and then calculated the adsorption energies *E*_ads_ of H at all of the symmetrically‐inequivalent surface sites. *E*_ads_ is related to the free energy of H adsorption $G_{H^{*}}$, which has previously been found to be a good proxy for HER activity.^[^ ^50^ ^]^ Specifically, an *E*_ads_ around −0.24 eV compensates for the loss of entropy from gas‐phase H_2_ and the gain in vibrational zero‐point energy of the adsorbed H atom to give $G_{H^{*}}$ ≈ 0, which avoids the kinetics of H adsorption or release being too slow. Full details of the calculations and analysis are given in the Supporting Information.

**Figure 5:**
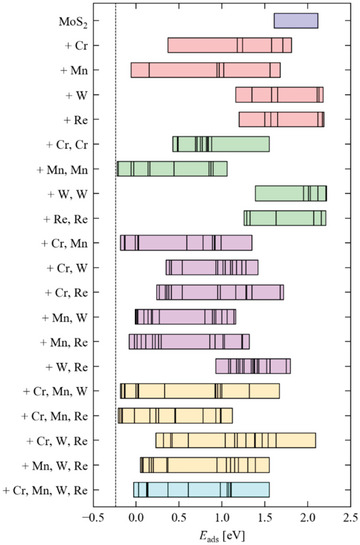
Range of H adsorption energies *E*_ads_ for an MoS_2_ surface slab model with up to four surface Mo atoms substituted with Cr, Mn, W, and Re calculated using density‐functional theory (DFT). The ideal *E*_ads_ = −0.24 eV, corresponding approximately to a thermodynamically‐neutral free energy of binding $G_{H^{*}}$,^[^ ^50c^ ^]^ is indicated by a black line.

**Figure** **5** shows the range of calculated *E*_ads_ for H adsorption at each of the unique surface sites in our MoS_2_ slab and each of the 19 substituted configurations (images of the bare surfaces and surfaces with bound H are provided in Figures S17–S37, Supporting Information). Our calculations predict a large *E*_ads_ of 1.61 eV for the basal‐plane S sites on pristine MoS_2_, which would result in slow H binding and poor HER activity. Calculations on the singly‐substituted surface models show that substitution with any of the four alloying elements activates these sites, with the predicted smallest *E*_ads_ for the Cr‐, Mn‐, W‐ and Re‐substituted surfaces being 0.37, −0.06, 1.16, and 1.20 eV respectively. We note that $G_{H^{*}}$ has an exponential relationship to the equilibrium constant for the H adsorption kinetics, so a small improvement in $G_{H^{*}}$ can have a large impact on the activity. Substitution with the first‐row transition metals (TMs) Cr and Mn in particular produces a large activation, and some of the models incorporating these elements are predicted to have binding sites with *E*_ads_ close to the −0.24 eV required for optimal H adsorption and release kinetics. For the models incorporating the first‐row TMs, the most active binding sites are invariably the neighboring S atoms whereas W and Re sometimes activate more remote surface S sites, which together produce an effect whereby larger numbers of substitutions generally lead to a higher density of more active surface sites.

We also examined the impact of the substitutions on the electronic structure of the slab models (see Figures S38–S57, Supporting Information). Barring the introduction of a small density of gap states in some models, the alloying metals generally produce a similar density of states to Mo such that the calculated electronic structure of the MoS_2_ slab is largely preserved. In particular, the positions of the valence‐ and conduction‐band levels of all of the substituted slabs are predicted to remain similar to the MoS_2_ slab, and for the models with larger numbers of substitutions, the Fermi energy *E*_F_ remains within ≈0.25 eV of that of the pristine MoS_2_ slab.

An additional point of note is that the calculations predict a small but not insignificant difference in energy between different arrangements of the substituting elements, ranging from 13 meV for the double substitution with W to 0.52 eV for the triple substitution with Cr, Mn, and Re. For (MoWReMnCr)S_2_, these enthalpic terms would likely be offset by the large configurational entropy, ensuring a homogenous distribution of metals at the surface site to maximize the activity of the basal‐plane S sites.

Overall, these calculations suggest that the high electrocatalytic performance of t(MoWReMnCr)S_2_ is likely due to activation of the basal plane toward H adsorption giving close to thermodynamically‐neutral H adsorption and release kinetics, while largely preserving the favorable electronic structure of MoS_2_. We note that it is also possible that the metal substitutions could improve the activity of the edge sites; while we did not investigate this in our calculations, we would expect the changes to the activity of the basal‐plane sites to have a more significant impact on the overall catalytic activity due to their larger surface area.

To experimentally elucidate the mechanistic aspects of HER, we generated Tafel plots from the corresponding polarization curves and the data are presented in Figure 4b. A decrease of ≈19 mV dec^−1^ is recorded for (MoWReMnCr)S_2_ (133.8 mV dec^−1^) compared to MoS_2_ (152.9 mV dec^−1^), which implies a change in the reaction mechanism. Considering the average overpotential region within which the Tafel slopes were calculated, the value of 133.8 mV dec^−1^ determined for the (MoWReMnCr)S_2_ catalyst lies close to the theoretical one (120 mV dec^−1^) reported for a Volmer‐limited HER process.^[^ ^51^ ^]^ This finding is in line with our DFT calculations presented above, predicting the activation of the basal plane of (MoWReMnCr)S_2_ toward H adsorption. The tuned adsorption energetics on the surface of (MoWReMnCr)S_2_ facilitate the Volmer step (which involves the formation of adsorbed hydrogen atom on the surface of the catalyst), a phenomenon depicted in the lower Tafel slope value for this material. The same trend is also observed upon mixing the catalysts with CB, which further highlights the significance of the beneficial effect of metal substitutions on the mechanism (and through that on the activity) of the catalyst (assuming a similar effect in the conductivity of the catalysts’ films after mixing with CB). The exchange current density, *j*_0_, was calculated by extrapolating the Tafel lines to 0 V and found to be 16.9 µΑ cm^−2^ for (MoWReMnCr)S_2_, which was significantly higher than the 2.67 µΑ cm^−2^ calculated for MoS_2_. Furthermore, upon mixing the as prepared pristine materials with 20% CB, *j*_0_ increases to 89.5 and 37.2 µΑ cm^−2^ for (MoWReMnCr)S_2_@20%CB and MoS_2_@20%CB, respectively, as expected from the increased conductivity of the catalysts. However, *j*_0_ values are only a rough approximation of the intrinsic activity of the catalysts because they are dependent on the real surface area of the electrodes and thus scale accordingly.

To provide further insight into the kinetics of the HER, we conducted electrochemical impedance spectroscopy (EIS) measurements on the (MoWReMnCr)S_2_@20%CB and MoS_2_@20%CB electrodes. From the qualitative features of the alternating current (impedance) data presented in Figure S8, Supporting Information, it can be deduced that two‐time constants, *τ*_RC_, are identified at the applied potential bias, that is, −0.47 V versus Ag/AgCl_(3.5 M KCl)_, which is most evident in the Bode phase plots in Figure S15c, Supporting Information). The high frequency *τ*_RC_ is ascribed to the intrinsic porosity of the electrode film as has been previously reported.^[^ ^52^ ^]^ Furthermore, due to the use of stationary electrodes in our study, the high rate of hydrogen gas evolution might introduce a concentration barrier on the surface of the electrode. This phenomenon will give rise to a finite diffusion‐type element that is expected to respond in the high‐to‐medium frequency range (see, for e.g., ref. [53] and references therein). Therefore, we can conclude that the first *τ*_RC_ is of geometric and/or diffusion origin; and thus, contains no information about the kinetics of the reaction. As the applied frequency decreases, the HER results in the flow of DC current, and hence, a second *τ*_RC_ is generated, being directly related to the kinetics of the reaction. To simulate the AC response of the system, we used a two‐Randles in series model (Figure S15d, Supporting Information) modified for the kinetic and time constant dispersion effects by replacing the ideal capacitors with constant phase elements (CPEs).^[^ ^52^, ^53^ ^]^ The first branch, denoted as *R*_d_ *CPE*_d_ is associated with the *τ*_RC_ of geometric/finite‐diffusion origin and the second one referred to as *R*_ct_ *CPE*_dl_ is associated with the kinetics of the HER. The most significant feature of merit derived from the analysis of the EIS data is the ≈eight times lower charge transfer resistance, *R*_ct_, of the (MoWReMnCr)S_2_@20%CB (3.12 Ω cm^2^) compared to that determined for the MoS_2_@20%CB electrode (25.11 Ω cm^2^, see Table S5, Supporting Information). This finding is in line with the direct current (voltammetric) data in Figure 4 and shows the significantly higher catalytic activity toward HER of the multicomponent catalyst.

We next attempted to assess the intrinsic activity of the (MoWReMnCr)S_2_@20%CB system by decoupling the surface‐area effects from the overall catalytic activity. A straightforward approach is to approximate the electrochemically‐active surface area (ECSA) of the electrode from the capacitance of the interface, *C*_dl_, because the latter is directly proportional to the former.^[^ ^54^ ^]^ As has been previously reported, *C*_dl_ can be estimated within the HER potential region using EIS data.^[^ ^52^ ^]^ However, in our case, the high degree of frequency dispersion effects recorded at the interface, depicted in the large deviation of the *CPE*_dl_ exponential factor, *n*_dl_, from unity, render this approach rather unreliable. To address this issue, an alternative strategy has been followed that involves the use of cyclic voltammetry (CV) experiments within a potential region where no faradaic reactions occur. In this way, the integral (average) capacitance, *C*_int_, of the interface can be approximated. The corresponding CV scans are presented in Figure S16a, Supporting Information, where a typical *pseudo*‐rectangular response is observed. The current density readings at 0.1 V versus RHE were extracted from the cyclic voltammograms and their dependence on the scan rate was examined (Figure S16d–f, Supporting Information). The current density is proportional to scan rate, as expected for a blocking interface, and thus *C*_int_ can be determined by the slope of the curves. We calculate the *C*_int_ to be 10.3 and 5.4 mF cm^−2^, respectively for the (MoWReMnCr)S_2_@20%CB and MoS_2_@20%CB electrodes. For reference, the same experiments are conducted on a pure CB electrode, for which the *C*_int_ is determined to be 0.22 mF cm^−2^. Subtracting this value from the overall capacitance determined for the catalyst/CB mixture, as the capacitive contributions are expected to behave in parallel, we obtain values of 10.08 and 5.18 mF cm^−2^ for the (MoWReMnCr)S_2_@20% CB and MoS_2_@20% CB electrodes. The ratio of the aforementioned *C*_int_ values is ≈1.95, implying an increased surface area for the (MoWReMnCr)S_2_@20%CB electrode compared to that of the MoS_2_@20%CB. Having estimated the ratio of *C*_int_ for both electrodes, we then assess the intrinsic catalytic activity of these catalysts by combining the AC and DC data. Assuming no significant clogging effects during the HER, the surface area effects can be decoupled from the values of *R*_ct_ by normalizing the latter with the obtained *C*_int_ values. The product *R*_ct_ *C*_int_ can be used as an indicator of the intrinsic activity of the catalysts in a similar way to current density per ECSA;^[^ ^54^ ^]^ the lower the value, the higher the intrinsic activity, or vice versa. We obtain *R*_ct_ *C*_int_ values of 31.45 and 130.07 ms for the (MoWReMnCr)S_2_@20% CB and MoS_2_@20% CB electrodes, respectively. This finding demonstrates that the high observed HER catalytic activity for the multicomponent catalyst is primarily attributed to the intrinsic activity of the material and is not solely to an increase in the ECSA. The same conclusion can be reached by comparing current density (per nominal area) values at a constant overpotential in the DC data in Figure 4a. For example, the ratio of the recorded current densities ((MoWReMnCr)S_2_ to MoS_2_) at *η* = −0.5 V is ≈7.8, which is significantly larger than the corresponding capacitance ratio of ≈1.95. This further emphasizes the more favorable intrinsic properties of the (MoWReMnCr)S_2_ catalyst compared to MoS_2_.

From a device‐development perspective, the chemical and electrochemical stability of an electrocatalyst is crucial to the overall performance of an electrolyzer cell. To test this, the activity of the (MoWReMnCr)S_2_@20%CB electrocatalyst was evaluated over longer timescales. Initially, a 7000‐cycle cyclic voltammetry (CV) test curve was measured, which indicated that the (MoWReMnCr)S_2_@20%CB is very stable within the timeframe of the measurement (Figure 4d). Furthermore, the stability of the (MoWReMnCr)S_2_@20%CB catalyst was further tested by medium‐term chronopotentiometry experiments over a 20 h period at a *j* of 10 mA cm^−2^, which confirmed the retention of catalytic activity after continuous operation for an extended period of time (Figure 4f).

## 3 Conclusion

In conclusion, we have demonstrated a facile low‐temperature approach toward the synthesis of the high entropy transition metal dichalcogenide  (MoWReMnCr)S_2_ by combining five individual single‐source precursors. The as‐synthesized (MoWReMnCr)S_2_ was exfoliated to obtain few‐layer 2D nanosheets with a homogeneous mixing of metals at the nanoscale. 2D (MoWReMnCr)S_2_ was evaluated as an electrocatalyst for the HER and was found to display significantly improved catalytic activity compared to the widely‐studied 2*H*‐MoS_2_. In particular, a (MoWReMnCr)S_2_@20%CB electrocatalyst blend incorporating conductive carbon black was found to require a much lower overpotential of 229 mV to reach a current density of 10 mA cm^−2^ compared to a potential of 362 mV for the comparable MoS_2_@20%CB electrocatalyst. This enhanced performance is suggestive of a synergistic effect from the presence of multiple cations within the system (i.e., the so‐called cocktail effect), which is supported by DFT calculations of substituted 2*H*‐MoS_2_ surface‐slab models showing significant activation of the basal‐plane sites toward H adsorption, particularly by Mn and Cr atoms. Our scalable synthesis approach is expected to be a universal route to the production of 2D HE materials of various transition‐metal disulfides. And in terms of fundamental science, the entropic stabilization of nanosheets is potentially a powerful approach to produce 2D materials that cannot otherwise be produced due to their intrinsic instability in the low‐dimensional limit.

## 4 Experimental Section

### Chemicals

Molybdenum hexacarbonyl Mo(CO)_6_ (98.0%), tetraethylthiuram disulfide (≥97%), ammonium tetrathiotungstate (≥99.99%), sodium diethyldithiocarbamate trihydrate (≥98%), sulfur (≥99.98%), ammonium sulfide (20 wt% in water), tetraethylammonium bromide (≥99%), ammonium perrhenate (≥99.0%), bis(diethylthiocarbamoyl)disulfide (≥97%), manganese(II) acetate tetrahydrate (≥99%), chromium(III) chloride hexahydrate (≥96%), and silica gel (40–63 µm particle size) were purchased from Sigma–Aldrich and used without further purification. Acetone (≥99.0%) dichloromethane (≥99.0%), methanol (≥99.5%), diethyl ether (≥99.0%), acetonitrile (≥99.9%), hexane (≥97.0%), dry acetonitrile (≥99.80%), pentane (≥99.0%), isopropanol (≥99.5%), and hydrochloric acid (37%) were purchased from Sigma–Aldrich and used as received.

### Synthesis of Bulk (MoWReMnCr)S_2_ Powders

An equimolar mixture of the dithiocarbamate precursors MoL_4_, WS(S_2_)L_2_, Re_2_(µ–S)_2_(L)_4_, MnL_3_, and CrL_3_ (L = S_2_CNEt_2_; synthesis details are given in the Supporting information) were placed in a ceramic boat in a Carbolite MTF 12/25/250 tube furnace and heated to 500 °C at a heating rate of 15 °C min^−1^ under argon flow (300 cm^3^ min^−1^) before being held at this temperature for 1 h. After cooling to room temperature, the sample was taken from the furnace and ground to a homogenous powder for 15 min using an agate mortar and pestle.

### Synthesis of Bulk MoS_2_

This material was prepared using the same method as the (MoWReMnCr)S_2_ powder, using only 50 mg of the MoL_4_ precursor as the starting material.

### Preparation of 2D (MoWReMnCr)S_2_ Nanosheets

Bulk (MoWReMnCr)S_2_ powder (10 mg) was added to N‐Methylpyrrolidone (NMP, 10 mL) in a centrifuge tube. The suspension was ultrasonicated for 24 h in an Elmasonic P bath operating at 37 kHz and 30% power, which was coupled with water‐cooling coil to keep the water temperature below 30 °C. The resulting dispersions were centrifuged at 1500 rpm using a Thermo Heraeus MultifugeX1 for 3 h to separate the exfoliated nanosheets from the remaining bulk material. After centrifugation, the transparent solution was collected for further characterization. For TEM, two drops of the dispersion were added to a copper Quanitfoil grid with a glass dropper pipette. The grid was thoroughly rinsed with 10 mL iso‐propyl alcohol (IPA) and 10 mL deionized water to remove surface NMP residue and dried in vacuo at 60 °C overnight. For AFM analysis, the suspension was further ultrasonicated for 1 h before being cast onto quartz substrates.

### Characterization

Powder X‐ray diffraction (PXRD) measurements were conducted on a PANalytical X'Pert Pro machine using Cu K*α* radiation (1.5406 Å), with zero‐background sample holders used to reduce noise, and lattice parameters were obtained via Rietveld refinement using the TOPAS software. Atomic force microscopy (AFM) was performed using a Bruker Multimode 8 AFM equipped with a SiO_2_ substrate in PeakForce QNM mode. Scanning electron microscopy (SEM) and energy‐dispersive X‐ray spectroscopy (EDX) were performed using a TESCAN MIRA3 SC + OI EDS with accelerating voltages of 10 and 20 kV, respectively. Raman spectroscopy was performed using a HORIBA LabRAM Evolution HR with a 488 nm excitation laser. High angle annular dark field–scanning transmission electron microscopes (HAADF‐STEM) images and bright field images were acquired using a Thermo Fisher Titan STEM (G2 80–200) equipped with a Cs probe corrector (CEOS). X‐ray energy dispersive scanning (EDX) elemental mapping was performed using a ChemiSTEM Super‐X EDX detector in STEM mode operated at 200 kV.

### Electrochemistry

All the electrochemical measurements were carried out using a Metrohm Auto‐Lab potentiostat PGSTAT302N equipped with the FRA32 module and Nova 1.11 software. A two‐compartment cell configuration was used comprising a Pt mesh counter electrode separated from the reference (Ag/AgCl (3.5 m KCl)) and working electrodes by a poly(vinyldifluoridene) membrane with pores of 100 nm diameter. This setup avoids contamination from the counter electrode, as previously reported in the literature.^[^ ^48^, ^55^ ^]^ A solution of 0.5 m H_2_SO_4_ purged with N_2_ for 20 min was used as the supporting electrolyte. To prepare the working electrode, the active material was mixed with carbon black Super P (Alfa Aesar, 99+%) in the ratios required to reach 20 wt%. The mixture was ultrasonically dispersed in a 4:1 v/v water–ethanol solution containing 0.04 wt% Nafion solution (Sigma–Aldrich, protonic form, 5% w/w solution in a mixture of lower aliphatic alcohols and 45% water) in order to obtain a homogeneous dispersion. The resultant ink was drop cast onto 3 mm diameter glassy carbon with catalyst loading of 0.6 mg cm^−2^ as a working electrode. Unless otherwise specified, all potentials measured were with respect to the reversible hydrogen electrode (RHE), following the relation *E*_RHE_ = *E*_Ag/AgCl_ + 0.059 pH + 0.205 V. The final potential was converted to RHE by adding a value of 0.222 V. A Pt (10% w/w)/C catalyst from Alfa Aesar was also used for comparison, with the electrode film made following the process described above. The electrocatalytic performance of the prepared materials toward HER was investigated via custom‐made experimental procedures integrated with the Nova software, similar to those reported previously.^[^ ^47^, ^48^ ^]^ In brief, a series of five consecutive electrochemical impedance spectroscopy measurements (EIS) was performed with a step potential of 50 mV in the potential range of −0.4 to −0.6 V versus Ag/AgCl(3.5 m KCl) over the frequency range of 100–50 kHz with an imposed AC RMS amplitude of 7 mV peak‐to‐peak. The uncompensated resistance, *R*_u_, was calculated from the data by averaging the real part of the total impedance at each potential pulse. Prior to any measurements, the working electrodes were cycled at least 50 times using a cyclic voltammetry (triangular) waveform. This was done to activate the entire surface of the catalyst material by removing any trapped air introduced during the preparation process. Linear‐sweep voltammetry (LSV) measurements were conducted at scan rate of 5 mV s^−1^ over a potential window of −0.2 to −0.7 V versus Ag/AgCl(3.5 m KCl). The Tafel slope was calculated to assess the HER kinetics of the catalyst using the Tafel equation *η* = *a* + *b* log(*j*), where *η*, *j*, and *b* are the overpotential, current density, and Tafel slope, respectively. EIS measurements were recorded at a constant potential of −0.47 V versus Ag/AgCl(3.5 m KCl) over a frequency range of 20 kHz to 100 mHz with an imposed RMS amplitude of 7 mV peak‐to‐peak. All EIS data were subjected to a Kramers–Kroning (K–K) test, and only data that complied with the K–K criteria (i.e., had relative residuals of less than 5% for both the real and imaginary parts of the impedance) were used for the EIS analysis. The theoretical AC response of the equivalent circuit was calculated in the frequency domain and its response fitted to the experimental data using the Solver tool in Microsoft Excel to minimize the sum of squared differences between the experimental and theoretical data. The moduli of the calculated impedances were used as weighting factors. The chi‐square parameter, indicative of the goodness of the fit, was on the order of 10^−3^ for all data. The electrochemical active surface area (ECSA) was estimated by calculating the double‐layer capacitance of the electrodes from cyclic voltammetry data for various scan rates from 10–100 mV s^−1^ in the potential region of 0.02–0.18 V versus RHE. The medium‐term stability of the prepared material was evaluated by continuous potential cycling over a potential window between −0.2 and −0.6 V versus Ag/AgCl(3.5 m KCl) at a scan rate of 200 mV s^−1^ for 7000 cycles. Polarization curves were recorded, based on the approach described above, before and after the CV experiment, and compared. For long‐term stability tests, chronopotentiometry was performed at 10 mA cm^−2^ for 20 h, with the electrode rotated at 1000 rpm to decrease bubble formation over the electrode surface within the experimental timeframe.

## Conflict of Interest

The authors declare no conflict of interest.

## Supporting information

Supporting Information

Supporting Information

Supporting Information

## Data Availability

The data that support the findings of this study are available from the corresponding author upon reasonable request.
