# Supplementary material for: A Low‐Temperature Synthetic Route Toward a High‐Entropy 2D Hexernary Transition Metal Dichalcogenide for Hydrogen Evolution Electrocatalysis
Source: Adv Sci (Weinh). 2023 Mar 23;10(14):2204488. doi: 10.1002/advs.202204488 (PMC10190663; doi:10.1002/advs.202204488)
Supplement: Supplementary file 1 — Supporting Information [file ADVS-10-2204488-s002.pdf]

Supporting Information for:
A low-temperature synthetic route towards a high-entropy 2D hexernary transition metal dichalcogenide for hydrogen evolution electrocatalysis

*Jie Qu, Amr Elgendy, Rongsheng Cai, Mark A. Buckingham, Athanasios A. Papaderakis, Hugo de Latour, Kerry Hazeldine, George F. S. Whitehead, Firoz Alam, Charles Smith, David J. Binks, Alex Walton, Jonathan M. Skelton, Robert A. W. Dryfe, Sarah J. Haigh and David J. Lewis**

J. Qu, M. A. Buckingham, D. J. Lewis

Department of Materials

The University of Manchester, Oxford Road, Manchester, M13 9PL, UK.

E-mail: david.lewis-4@manchester.ac.uk

R. S. Cai, H. d. Latour, S. J. Haigh,

Department of Materials, National Graphene Institute and Sir Henry Royce Institute

The University of Manchester, Oxford Road, Manchester, M13 9PL, UK.

A. Elgendy, A. A. Papaderakis, R. A. W. Dryfe

Department of Chemistry and Sir Henry Royce Institute

The University of Manchester, Oxford Road, Manchester, M13 9PL, UK.

K. Hazeldine, A. Walton

Department of Chemistry and the Photon Science Institute,

The University of Manchester, Oxford Road, Manchester, M13 9PL, UK.

C. Smith, D. Binks

Department of Physics and Astronomy and the Photon Science Institute,
The University of Manchester, Oxford Road, Manchester, M13 9PL, UK.

F. Alam, J. M. Skelton
Department of Chemistry
The University of Manchester, Oxford Road, Manchester, M13 9PL, UK.

F. Alam
Present address: Department of Electronic and Electrical Engineering,
University College London, London WC1E 6BT, U.K.

Contents:

Synthesis of precursors

Figure S1: Proposed synthetic pathway for $Mn_2O_2(DTC)_4$

Figure S2. Crystal structure of the Mn precursor $Mn_2O_2(DTC)_4$ used throughout this report.

Figure S3. IR data of pure $Mn(DTC)_3$ and $Mn_2O_2(DTC)_4$

Figure S4. TGA data of Mn(DTC)3 and $Mn_2O_2(DTC)_4$ as a comparison.

Figure S5: Chemical structures of the precursors

Figure S6. FT-IR spectra of (a) CrL_3, (b) MoL_4, (c) $Re_2(l\text{-}S)_2(L)_4$, (d) $WS(S_2)L_2$

Table S1: Table of reported synthetic procedures for HE materials

Figure S7: SEM-EDX analysis of the bulk $(MoWReMnCr)S_2$ powders

Table S2: Elemental compositions of the HEDS material determined using SEM-EDX

Figure S8. Figure showing XPS fitting for MoS_2

Figure S9. High resolution XPS spectra

Figure S10. XPS survey spectra for bulk MoS_2 and $(MoWReMnCr)S_2$ powders

Figure S11. High resolution HAADF STEM image of a bilayer MoS_2 flake with monolayer edges.

Figure S12. Atomic model and HAADF STEM image of MoS_2 1T' phase

Figure S13. AFM images of exfoliated 2D flakes $(MoWReMnCr)S_2$

Figure S14: STEM-EDX analysis of the exfoliated 2D $(MoWReMnCr)S_2$ flakes

Table S3 & S4: Elemental composition determined using STEM-EDX

Figure S15: EIS spectra of the MoS_2@20% CB, 2D HEDS, and HEDS@20% CB electrodes

Table S5: EIS parameters calculated from CNLS fitting

Figure S16: Electrochemical double-layer capacitance of the MoS_2@20% CB and HEDS@20% CB electrodes

Table S6: Comparison oft he HER performance of previously-reported MoS_2-based materials and the HES@20% CB material in an acidic medium

Table S7: Summary of the surface-slab models examined in the modelling study

Figure S17: Optimised structures of bulk 2*H* MoS_2 and the five-layer (001) surface slab model

Figure S18: Optimised pristine MoS_2 surface slab and optimised slabs with H atoms adsorbed at the two unique surface sites

Figure S19-37: Optimised lowest-energy metal configurations and H binding configurations of the 19 substituted surfaces examined in the modelling

Figure S38: Electronic structure of the MoS_2 slab in the vicinity of the Fermi energy

Figure S39-57: Electronic structure of the lowest-energy metal configurations of the 19 substitued surfaces examined in the modelling

Figure 58: Comparison of the electronic structures of the pristine MoS_2 and substituted surface slabs with the energies referenced to the average 1s core level of the nine Mo atoms in the central layer of the slab

Synthesis of MoL_4 ($L=S_2CNEt_2$) (1)

Tetrakis(diethyldithiocarbamato)-molybdenum (**Figure S1(a)**) was synthesised following a previously-reported procedure.[1] Briefly, molybdenum hexacarbonyl (1.0 g, 3.7 mmol) and tetraethylthiuram disulfide (2.2 g, 7.6 mmol) were mixed in 40 ml of acetone and the resulting mixture refluxed at 80 °C for 2 h in a round-bottom flask. After naturally cooling to room temperature a black precipitate formed, which was subsequently collected by vacuum filtration. The solid was thoroughly washed with pentane (3 × 30 mL) to collect the title compound, which was then dried at 60 °C in vacuum oven overnight. Anal. calc. for MoL_4 (%): C, 34.86; H, 5.85; N, 8.13. Found (%): C, 34.74; H, 5.92; N, 8.03.

Synthesis of $[WS(S_2)(L)_2]$ (2)

This complex (**Figure S1(b)**) was synthesised following a previously-reported procedure.[1] Ammonium tetrathiotungstate (1.91 g, 5.42 mmol) and sodium diethyldithiocarbamate (5 g, 21.85 mmol) were dissolved in deionized water. Diluted HCl (2 M) was added dropwise until the pH of the solution was reduced to 2. The dark-green precipitate was collected by vacuum filtration and washed with 500 ml of deionized water. The crude complex was then dissolved, filtered and washed with acetone, and the solvent removed under vacuum before drying overnight in a vacuum oven. Anal. calc. for $WS(S_2)L_2$ (%): C, 20.83; H, 3.50; N, 4.86. Found (%): C, 20.42; H, 3.38; N, 4.62.

Synthesis of $[Re_2(\mu\text{-}S)_2(L)_4]$ (3)

This complex (**Figure S1(c)**) was synthesised following a previously-reported literature procedure,[2] which follows two parts. Firstly, ammonium sulfide (100 mL) was used to dissolve elemental sulfur (0.7 g, 20 mmol) and the solution stirred for 10 minutes. 6.3 g (30 mmol) tetraethylammonium bromide and 3.9 g (15 mmol) of ammonium perrhenate (NH_4ReO_4) were then added and the reaction stirred for 18 h at room temperature. The product was collected by vacuum filtration and washed with a mixture of deionized water,

ethanol, methanol, and diethyl ether in a ratio of 3:1:3:2. The product was dried in a vacuum oven. This crude product (2 g) was then recrystallized from a mixture of acetonitrile and toluene and cooled very slowly to room temperature to yield dark-green crystals, which were isolated by vacuum filtration and washed with a mixture of toluene and diethyl ether. The obtained product was dried in vacuum oven overnight. Anal. calc. for $(Et_4N)ReS_4$ (%): C, 21.62; H, 4.54; N, 3.15. Found (%): C, 21.42; H, 4.78; N, 3.21.

This complex was then utilised to make $[Re_2(l\text{-}S)_2(S_2CNEt_2)_4]$ as follows. 1.1 g of $[(Et_4N)ReS_4]$ and 0.999 g of bis(diethylthiocarbamoyl)disulfide were reacted in 50 mL of dry acetonitrile for 24 h. The green precipitate was collected by vacuum filtration, washed with a mixture of CH_2Cl_2 and hexane and dried in a vacuum oven overnight to obtain the final product. Anal. calc. for $[Re_2(l\text{-}S)_2(S_2CNEt_2)_4]$ (%): C, 23.35; H, 3.92; N, 5.45. Found (%): C, 23.89; H, 4.08; N, 5.56.

A note on oxidation of the Mn precursor

The Mn precursor was initially synthesised using 2 equivalents of diethyldithiocarbamate ligands to balance the charge of the starting Mn salt. The product was analysed with elemental analysis and found to fit with the anticipated $Mn(DTC)_2$ product. However, this species rapidly oxidises to $Mn(DTC)_3$.[1–3] Indeed, many synthesis methods have been reported which report the use of $Mn(DTC)_2$, but we would like to point out that these have been wrongly reported and are likely to be $Mn(DTC)_3$ in fact.[3] In our study, elemental analysis was found to fit $Mn(DTC)_2$, which did not align with literature, in particular the studies by Eagle *et al* and Hendrickson *et al*.[1,2] This prompted further investigation into the exact complex which we made. The magnetic susceptibility (μ_{eff}) was measured. The ‘$Mn(DTC)_2$’ was found to have a μ_{eff} of 5.34 BM. This value is far from the expected value for tetragonal $Mn(DTC)_2$ of 5.92 BM or oxtahedral $Mn(DTC)_3$ 4.9 BM for the expected high spin complexes, respectively, indicating that this is not the species present.

Crystals grown and solved were found to be a new species with an $[O_2]^{2-}$ bridging dimer (Figure S2), this appears to inadvertently be the originally synthesised (and pure) ‘$Mn(DTC)_2$’

as the elemental analysis results are found to be an excellent fit: Found: C: 34.3%, H: 5.6%, N: 7.4%. Expected for $Mn_2O_2(DTC)_4 \cdot 0.75(CH_3)_2CO$: C: 34.3%, H: 5.7%, N: 7.2%. The proposed synthetic mechanism for formation of this species is shown below (Figure S1).

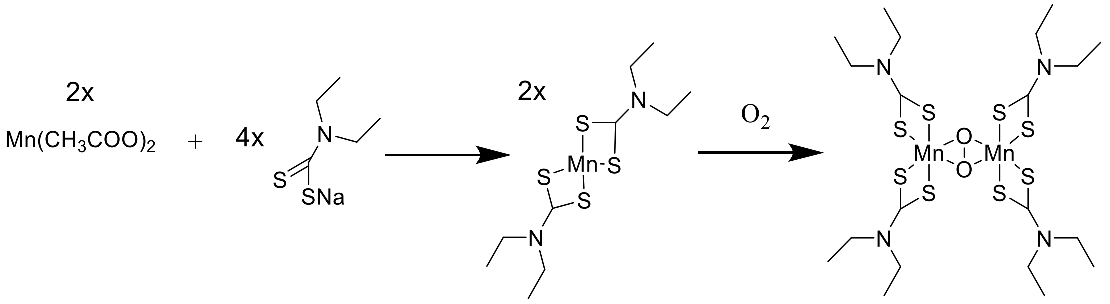

Figure S1. Proposed synthetic pathway for $Mn_2O_2(DTC)_4$.

Synthetic procedure for $[Mn_2O_2(DTC)_4]$

Sodium diethyldithiocarbamate (5 g, 21.85 mmol) and $Mn(CH_3COO)_2 \cdot 4H_2O$ (4.35 g, 30.83 mmol) were dissolved in deionized water (300 mL). After being filtered and washed with deionized water (3 × 100 mL), a black precipitate was formed which was recrystallized from acetone. This synthesis yielded a $Mn(DTC)_2$ dimer species with a bridging O_2 as shown in Figure S3. Mass spec found the $Mn(DTC)_2$ fragments (m/z = 350.99). Elemental analysis for was found to be (expected for $Mn_2O_2(DTC)_4 \cdot 0.75\ (CH_3)_2CO$) C: 34.3 (34.3), H: 5.6 (5.7), N: 7.4 (7.2). This precursor was used throughout this manuscript.

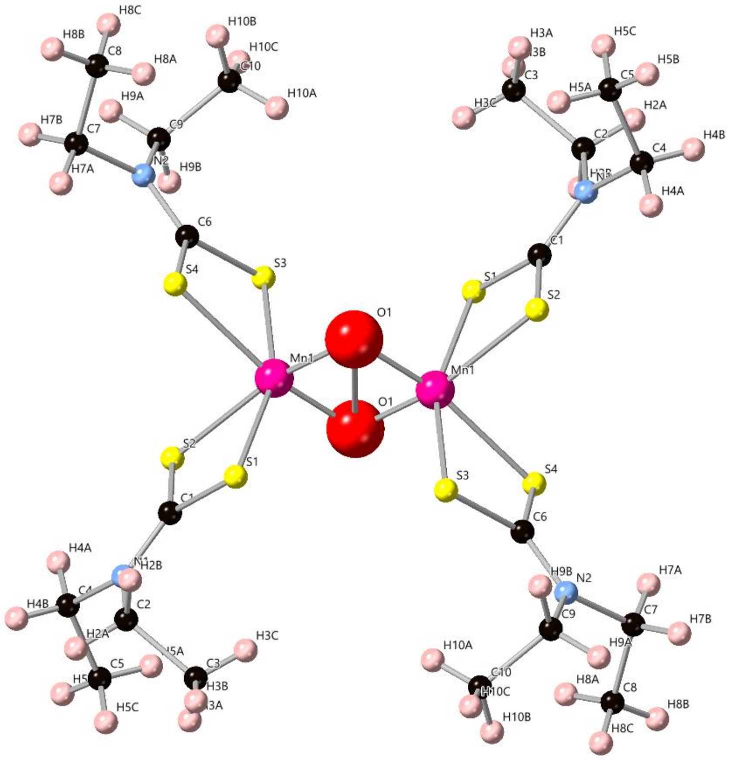

Figure S2. Crystal structure of the Mn precursor $Mn_2O_2(DTC)_4$ used throughout this report.

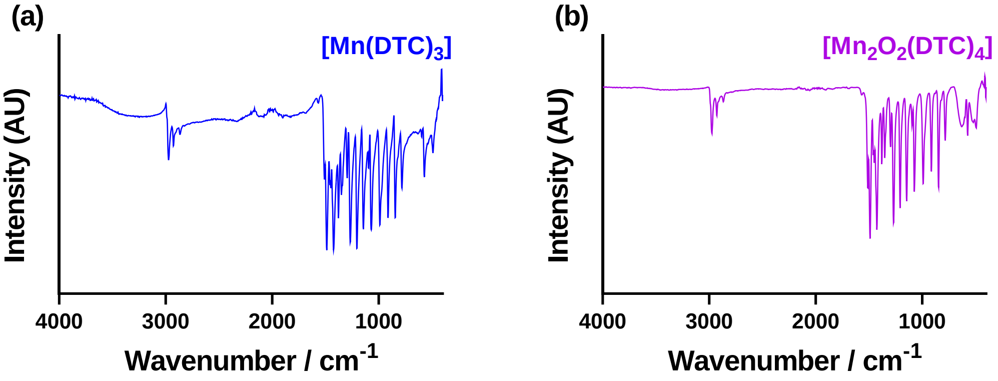

Figure S3. IR data of (a) pure $Mn(DTC)_3$: 571, 596, 782, 844, 912, 988, 1069, 1094, 1143, 1205, 1266, 1295, 1348, 1377, 1424, 1440, 1457, 1487, 1509, 2865, 2926, 2971. (b) $Mn_2O_2(DTC)_4$: 493, 519, 573, 626, 784, 845, 914, 990, 1073, 1095, 1145, 1207, 1268, 1297, 1350, 1378, 1426, 1448, 1456, 1489, 1510, 2866, 2928, 2973.

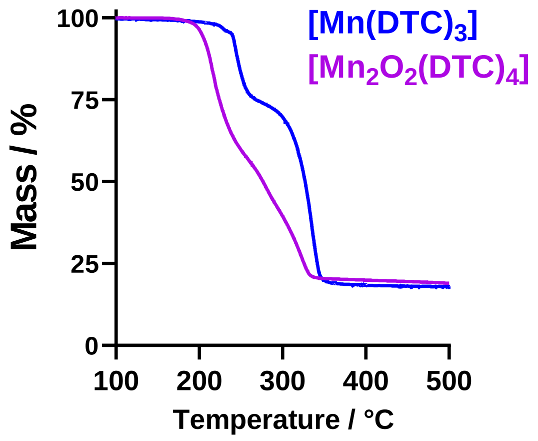

Figure S4. TGA data of Mn(DTC)3 and Mn2O2(DTC)4 as a comparison.

Synthesis of CrL_3 (5)

Tris(diethyldithiocarbamato) chromium (**Figure S1(e)**) was synthesised following a previously-reported literature procedure.[4] Chromium trichloride hexahydrate (2.0 g, 9 mmol) was dissolved in deionized water (250 mL) to form a green solution. The monosodium salt of diethyl dithiocarbamate (12.0 g, 54 mmol) was added in the solution to generate a blue precipitate, which was collected by vacuum filtration to obtain crude CrL_3. The crude product was purified using column chromatography on silica, eluting with dichloromethane or chloroform, and collecting the rapidly-eluted blue band. A vacuum evaporator was used to isolate the ultramarine solid from the solvent before drying in vacuum oven overnight. Anal. calc. for CrL_3 (%): C, 36.3; H, 6.1; N, 8.5. Found (%): C, 37.8; H, 6.6; N, 8.1.

Precursor Synthesis and Characterization:

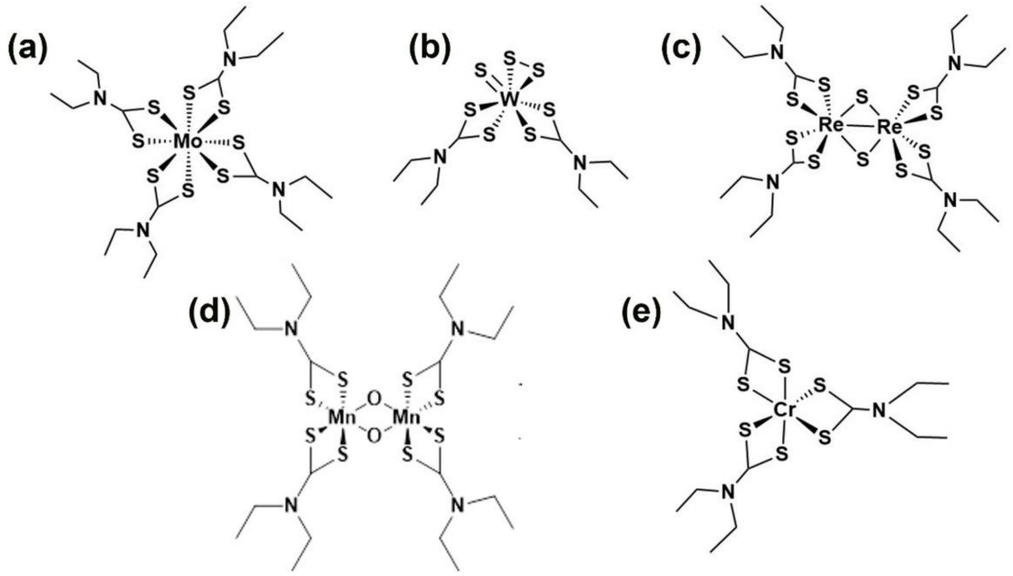

Figure S5. Chemical structures of the molecular precursors prepared in this work: (a) MoL_4, (b) $WS(S_2)L_2$, (c) $Re_2(\mu\text{-}S)_2(L)_4$, (d) $Mn_2O_2L_4$, and (e) CrL_3 ($L=S_2CNEt_2$).

FTIR spectra of the single-source precursors

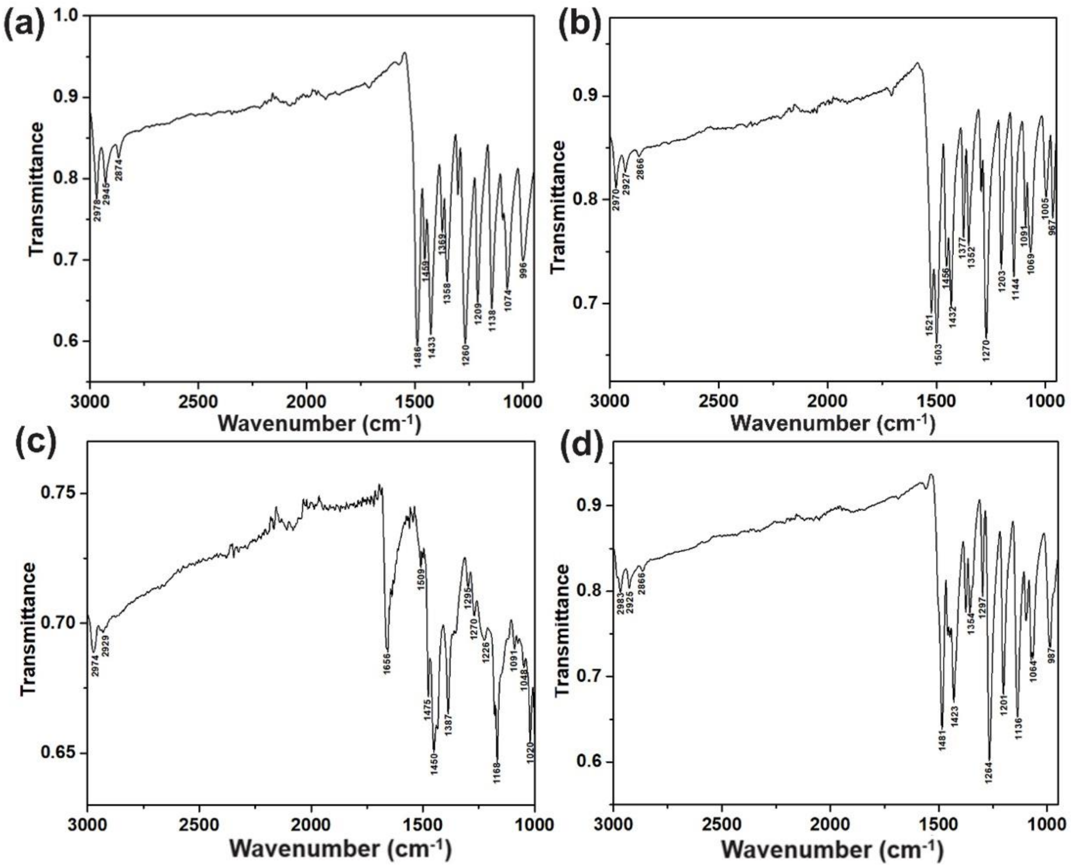

Figure S6. FT-IR spectra of (a) CrL_3, (b) MoL_4, (c) $Re_2(l\text{-}S)_2(L)_4$, (d) $WS(S_2)L_2$

Table of reported synthetic procedures for HE materials:

Table S1. Table of reported synthetic methods of producing HE chalcogenides, explaining the method, temperature and time of each reaction.

HE Materials	Synthetic Method	Reaction Temperature (℃)	Reaction Time (h)	Ref.
(ZnCoCuInCa)S	Multi-cation exchange	600	24	[5]
(MoWNbVTa)S_2	HF etching followed by elemental annealing	1000	24	[6]
$Cu_5SnMgGeZnS_9$	Ball-milling of elements followed by sintering	750	20	[7]
(GeSnPb)(SSeTe)	Elemental Annealing	1000	20	[8]
(PbSbSn)(SSeTe)	Elemental Annealing	1150	7	[9]
(TiVCrNbTa)S_2	Elemental Annealing and chemical vapor transport (CVT) method	1050	720	[10]
(TiVCrNbTa)Se_2		1000	336	
$(Ti,V,Cr,Nb)_{0.8}(Fe,Mn)_{0.2}Se_2$		1000	48	
(Ti,V,Zr,Nb,Hf)Te_2		1000	336	
(MoWReMnCr)S_2	Single source precursor decomposition	500	1	This work

SEM-EDX Analysis and Elemental Composition:

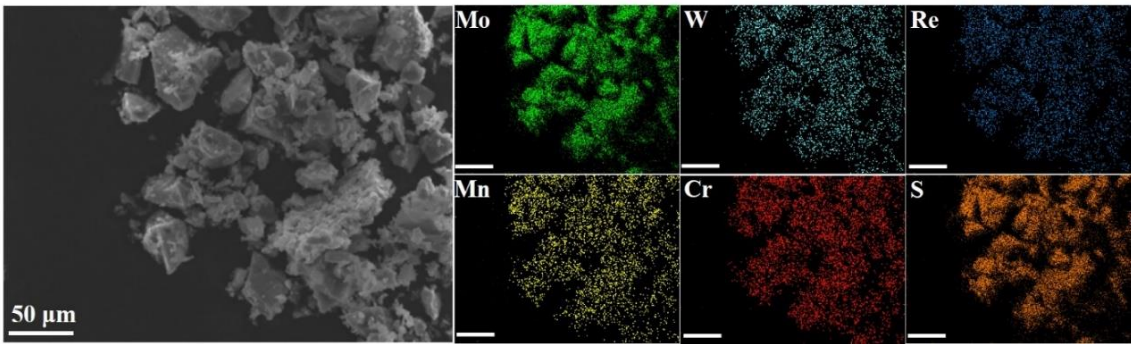

Figure S7. SEM-EDX maps showing the distribution of the elements present in bulk $(MoWReMnCr)S_2$ powders at an accelerating voltage of 20 keV. The far left image is the secondary electron image, while the smaller images show the distribution of Mo Lα, W Lα, Re Kα, Mn Kα, Cr Kα and S Kα emissions. All scale bars represent 50 µm.

Table S2. Elemental composition determined using SEM-EDX.

Elements	Mol % (nominal)	Mol % (found)
Mo	6.67	8.28
W	6.67	6.72
Re	6.67	5.34
Mn	6.67	4.95
Cr	6.67	4.71
S	66.7	69.9

[a] The Aztec software was used to quantify the relative concentrations of each element.

X-Ray Photoelectron Spectroscopy:

The Mo 3d spectra (**Figure S8(a) & S9(a)**) show three chemical species, which can be attributed to Mo^{4+} from MoS_2 and Mo^{6+} from MoO_3.[11] The third chemical species is attributed to another sulfide environment. It is noteworthy that the peaks at 232.8 eV (MoO_3) and 229.6 eV (MoS_2) in MoS_2 shifted to 232.2 eV and 228.6 eV, respectively in the high-entropy disulfide (HEDS). This shift indicates that the chemical environment of Mo is altered by the introduction of the other metals.[12] The fitted S 2p spectra (**Figure S8(b) & S9(b)**) show signals from sulfide (MoS_2) at 162.4 eV, and $[SO_4]^{2-}$ at 168.6 eV, respectively, which is consistent with previous reports on MoS_2.[11a, 13] Again, an additional sulfide environment could be observed which is consistent with the additional sulfide species observed in the Mo 2p spectra. In the HE MoS_2 analogue, the spectra could be deconvoluted into a single sulfate environment at 167.8 eV and three sulfide environments. The multiple sulfide environments can be attributed to the different metal-S bonding environments that are present in the system (through the presence of six different metals in our system) (**Figure 9(b)**). The two characteristic peaks at 653.4 and 641.8 eV in the Mn 2p spectra (**Figure S9(c)**) are attributed to the Mn $2p_{1/2}$ and $2p_{3/2}$ species, respectively.[15] Similarly, Cr $2p_{1/2}$ and $Cr2p_{3/2}$ species were observed in the Cr 2p spectra (**Figure S9(d)**) with peaks at 584.2 and 575.3 eV, respectively.[16] The valence states of Mn and Cr could not be accurately quantified due to the complex chemical surroundings.[17] There are two characteristic doublet peaks in the W 4f spectra (**Figure S9(e)**). The peak at 32.2 eV can be attributed to W^{4+} from WS_2 and 35.2 eV to the W^{6+} of WO_3. The core-level peaks in the Re 4f spectrum (**Figure S9(f)**) are located at 41.4 eV for ReS_2,[14] and 48.8 eV for Re_2O_7. A further sulfide environment was found for Re at 42.3 eV. The presence of oxide species for most of the metals is not uncommon for metal-sulphide materials,[18] but to establish whether these species arise from surface oxidation, or whether the oxides are present as bulk materials, additional characterization techniques were used.

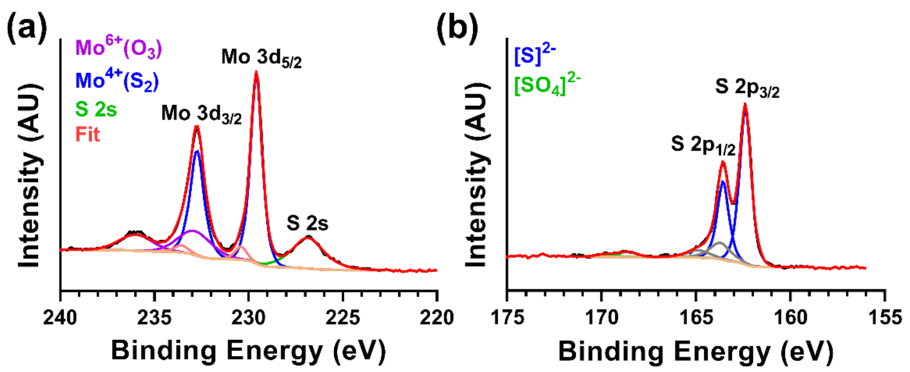

Figure S8. Figure showing XPS fitting for MoS_2

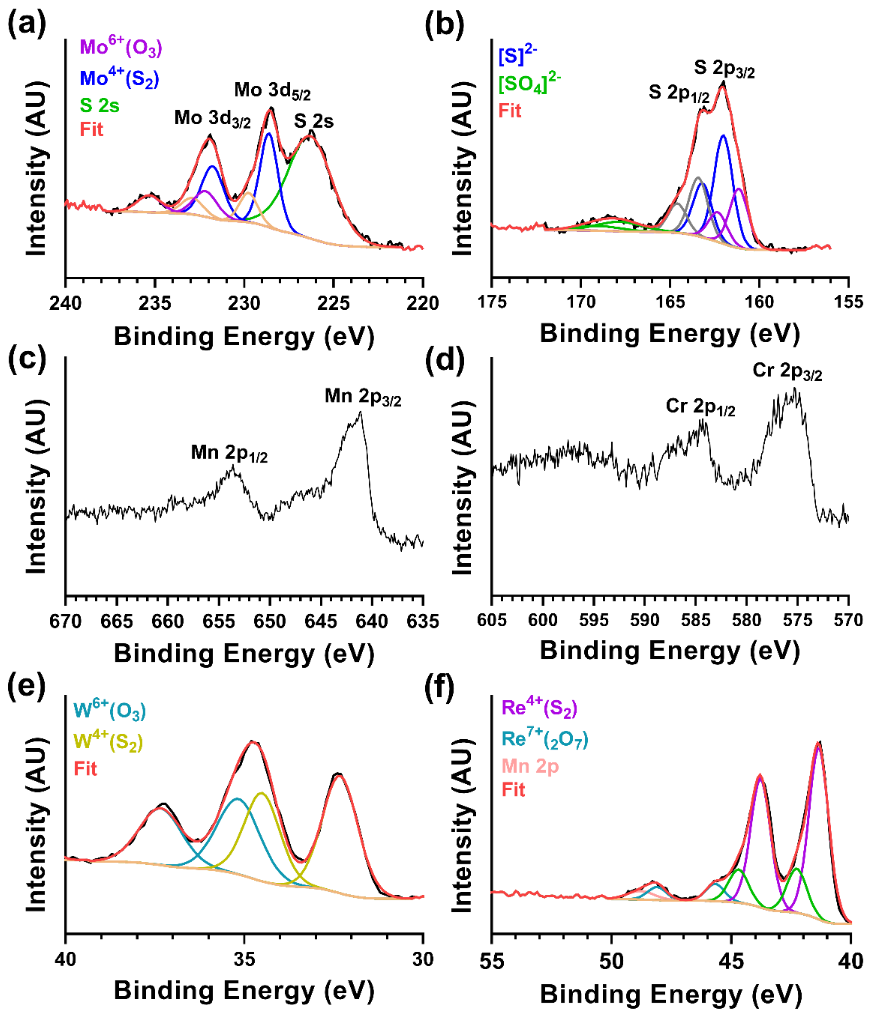

Figure S9. High resolution XPS spectra: (a)/(b) Mo 3d and S 2p spectra for $(MoWReMnCr)S_2$ and MoS_2. (c)-(f) W 4f, Re 4f, Mn 2p and Cr 2p spectra for $(MoWReMnCr)S_2$.

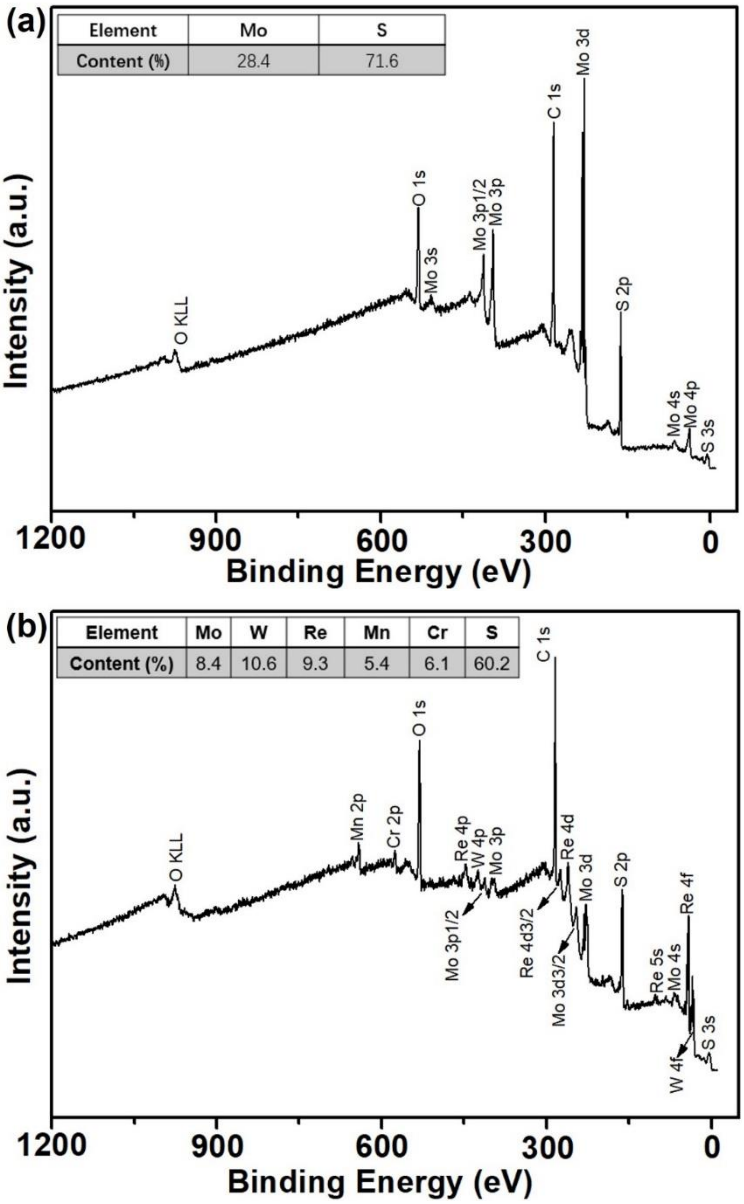

Figure S10. XPS survey spectra for bulk MoS2 and (MoWReMnCr)S_2 powders

Adventitious carbon and oxygen contaminants are usually observed on samples that have been exposed to air, however the O 1s peak could also result from a minor amount of surface oxidation in air of the as-prepared samples. The C 1s peak at 284.8 eV was used to calibrate the binding energy scale.[2, 18]

High resolution HAADF STEM

During our extensive High resolution HAADF STEM assessment of the high entropy (MoWReMnCr)S_2 we observed some monolayered material which can be attributed to the hexagonal 2*H* phase (Figure SX below). During this assessment it was also apparent that some 1*T*' had also been observed, although this was not present in either the pXRD or Raman analysis. Therefore we can conclude that this phase must be present as a very minor impurity.

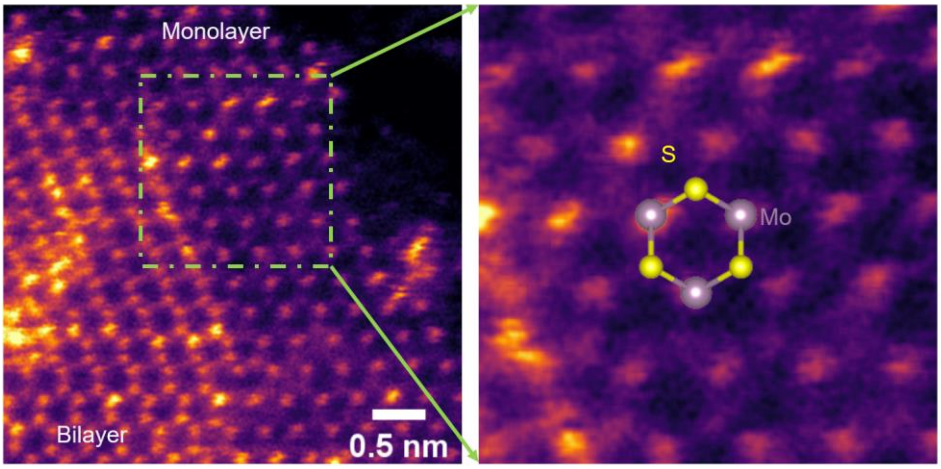

Figure S11. High resolution HAADF STEM image of a bilayer MoS_2 flake with monolayer edges.

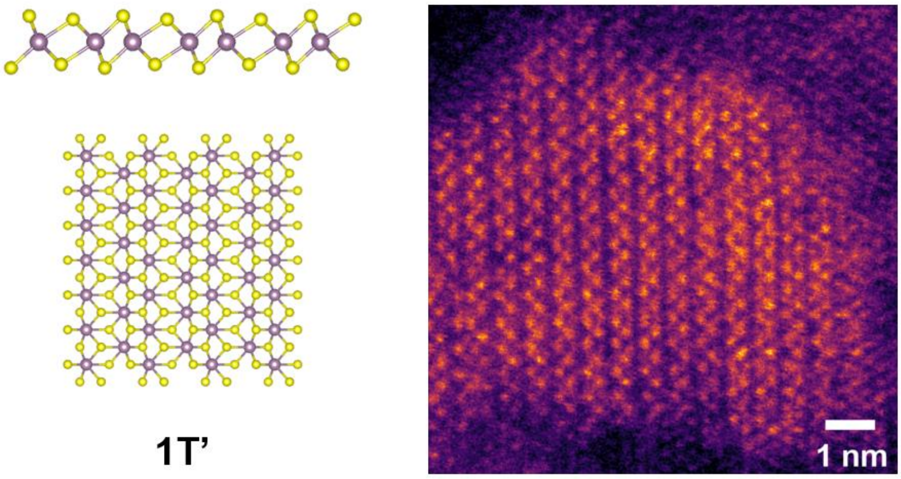

Figure S12. Atomic model and HAADF STEM image of MoS_2 1T' phase

Atomic Force Microscopy:

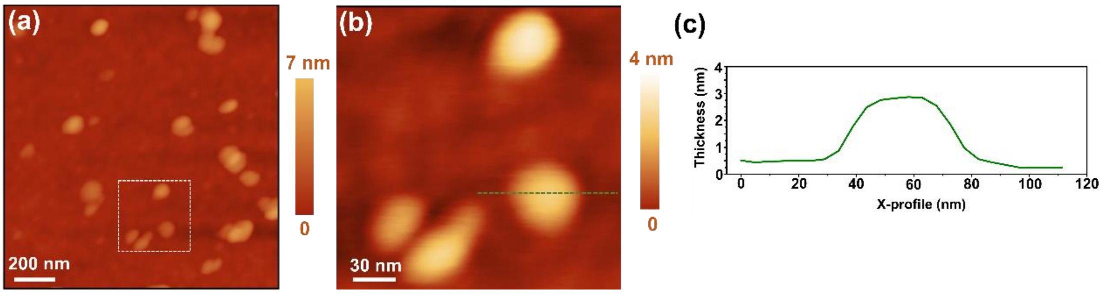

Figure S13. (a, b) AFM images of exfoliated 2D flakes $(MoWReMnCr)S_2$ with different magnifications and (c) The line scan plot of the labeled particle in (b).

STEM-EDX Analysis and Elemental Composition:

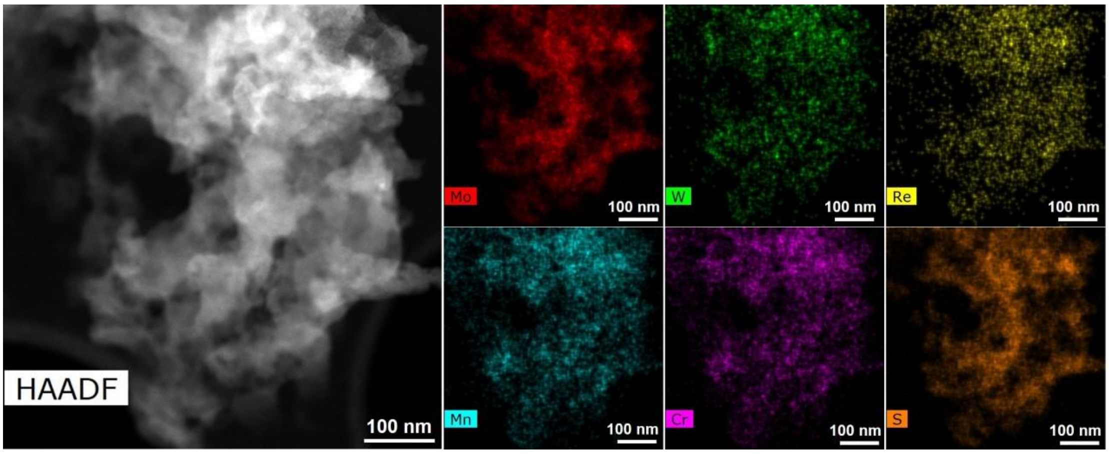

Figure S14. HAADF-STEM image of the 2D $(MoWReMnCr)S_2$ nanoflakes (left) and corresponding elemental maps of of the Mo Lα, W Lα, Re Lα, Mn Kα, Cr Kα and S Kα emissions (smaller images). All scale bars are 100 nm.

Table S3. Elemental composition determined using STEM-EDX by analysing the summed spectra for the area shown in **Figure S7**.

Elements	mol % (nominal)	mol % (found)
Mo	6.67	7.71
W	6.67	7.43
Re	6.67	5.83
Mn	6.67	4.95
Cr	6.67	4.28
S	66.7	69.8

Table S4. Elemental composition determined using STEM-EDX by analysing the summed spectra for the area in **Figure 4** in the main text.

Elements	Mol % (nominal)	Mol % (found)
Mo	6.67	7.98
W	6.67	6.94
Re	6.67	5.13
Mn	6.67	4.62
Cr	6.67	4.03
S	66.7	71.3

Electromchemical Analysis and Characterization:

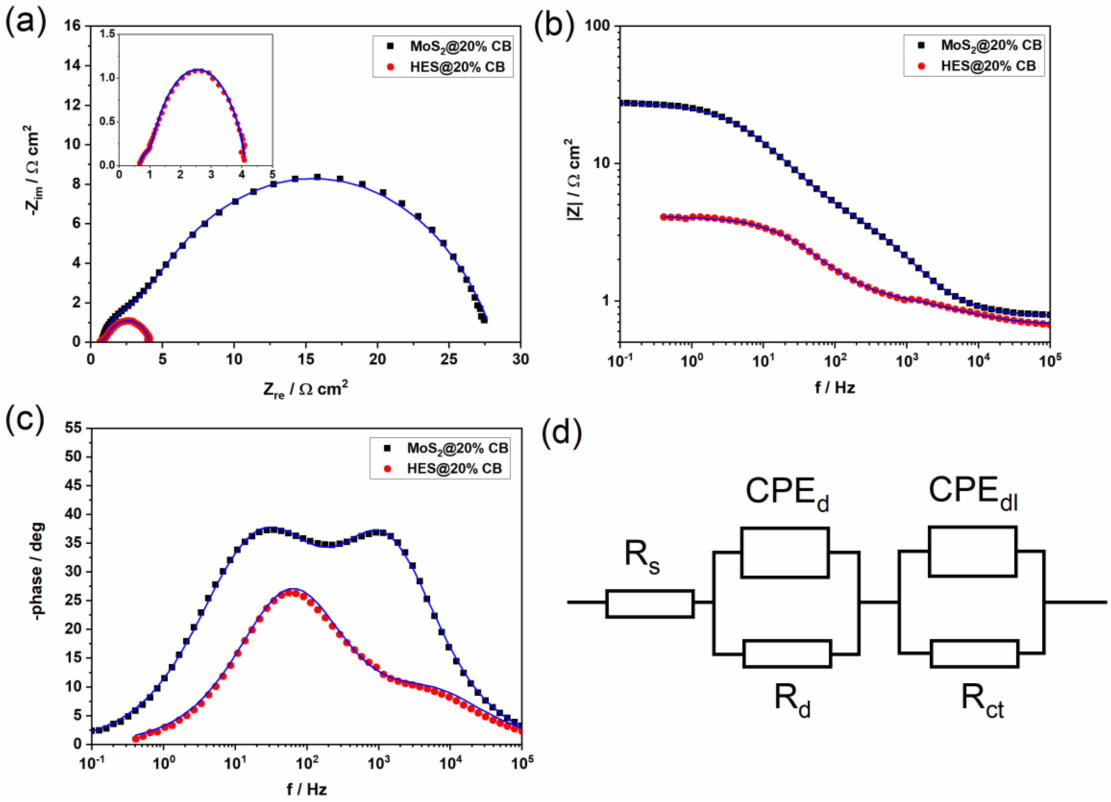

Figure S15. Electrochemical impedance spectra of the MoS_2@20% CB and HES@20% CB electrodes, adopting the (a) Nyquist, (b) Bode magnitude and (c) Bode phase representations. Points and continuous lines show the measured and simulated spectra, respectively. The inset in panel (a) shows a magnification of the HES@20% CB spectrum. (d) The two-Randles connected in series equivalent circuit used to simulate the AC response of the electrodes.

Table S5. EIS parameters calculated from CNLS fitting of the total impedance equation

for the two-Randles in series equivalent circuit model presented in **Figure S8(d)**.

Sample	$R_s(\Omega cm^2)$	$T_d\ (\Omega^{-1} s^{n_d} cm^{-2})$	n_d	$R_d(\Omega cm^2)$	$\tau_{dl}\ (\Omega^{-1} s^{n_{dl}} cm^{-2})$	n_{dl}	$R_{ct}(\Omega cm^2)$
MoS_2@ 20% CB	0.78	5.84×10^{-4}	0.83	2.32	2.86×10^{-3}	0.74	25.11
HEDS@ 20% CB	0.67	1.68×10^{-3}	0.73	0.33	6.32×10^{-3}	0.78	3.12

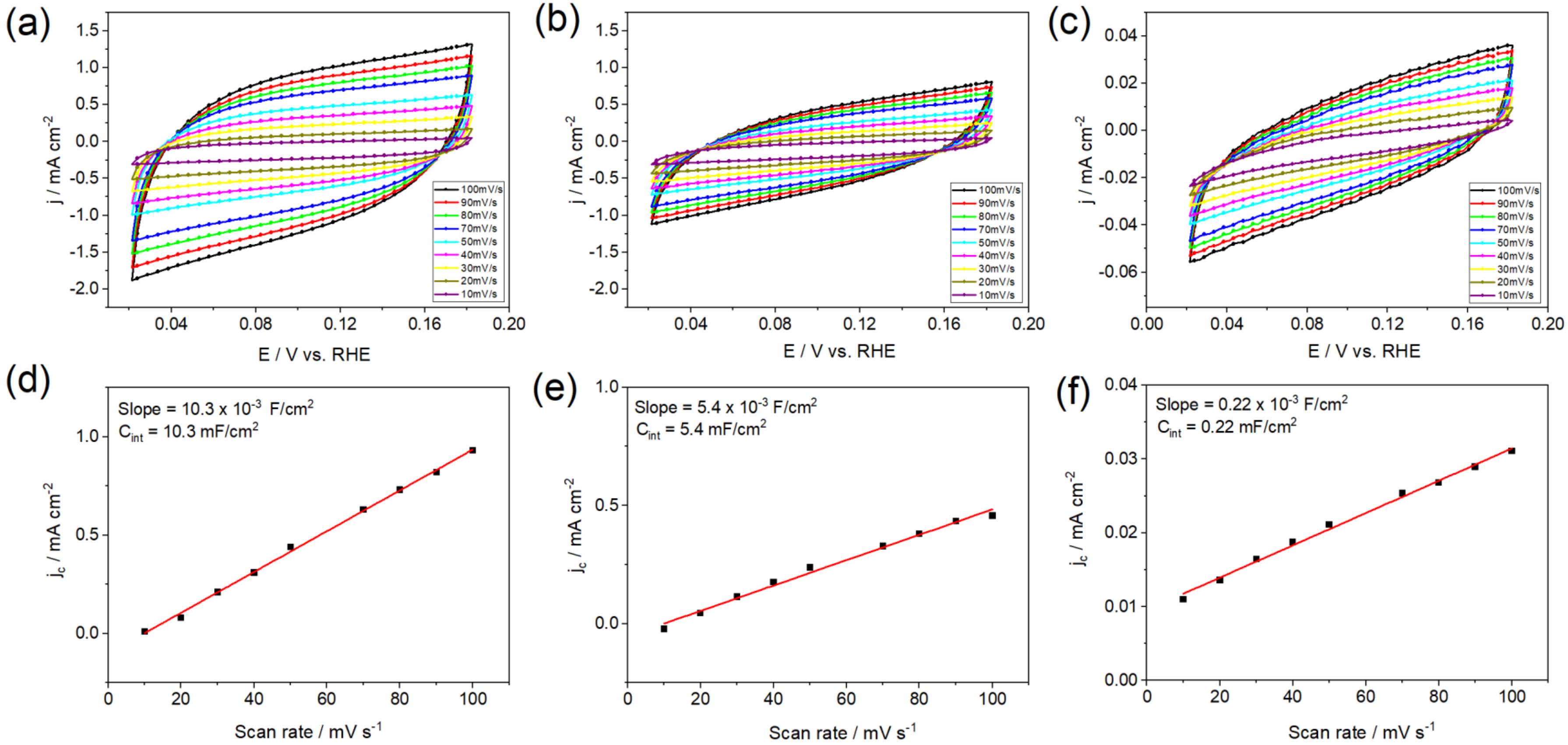

Figure S16. (a)-(c) Cyclic voltammograms for the HES@20% CB, MoS_2@20% CB and pure CB electrodes in the potential region between 0.02-0.18 V *vs*. RHE at scan rates from 10-100 mV s^{-1}. (d)-(f) Estimated electrochemical double-layer capacitance, C_{dl}, for the HES@20% CB, MoS_2@20% CB and pure CB electrodes calculated by plotting the capacitive current, j_C, at 0.1V *vs*. RHE against the scan rate.

Comparison of HER Performance to Previously-Reported MoS_2-Based Systems:

Table S6. Comparison oft he HER performance of previously-reported MoS_2-based materials to the synthesied HES@20% CB in an acidic medium.

Sample	η_{10} (mV vs. RHE)	Reference
Vertically Aligned MoS_2 Thin Films	−440	[19]
AuNP@MoS_2	−323	[20]
Au-MoS_2	−250	[21]
Nanostructured MoS_2	−430	[22]
MoS_2/SnS_2 heterostructure	−288	[23]
Glassy carbon@MoS_2	−266	[24]
Pore-rich monolayer MoS_2	−241	[25]
1*T*-MoS_2	−262	[26]
Au_{11}@MoS_2	−292	[27]
Ni-MoS_2	−302	[28]
WS_2/carbon cloth	−321	[29]
W_2C@WS_2 nanoflowers	−305	[30]
N-enriched C/WS_2 nanoflakes	−266	[31]
2H-WS_2	−500	[32]
1T-WS_2	−380	[33]
Edge-enrich ReS_2	−246	[34]

Carbonized wood/ReS_2	−260	[35]
1%Ce-ReS_2	−306	[36]
1T-$Mo_{0.85}W_{0.15}S_2$	-292	[37]
1T/2H-$Mo_{0.94}Cr_{0.06}S_2$	-250	[16]
Mn-doped MoS_2	-318	[15]
$(MoWReMnCr)S_2$	-229	This work

Density Functional Theory Calculations

To investigate the impact of alloying on the catalytic activity of the HE MoS_2 nanoparticles, we performed density-functional theory (DFT) calculations on a series of slab models of the basal plane (001) surface. The 2H structure of bulk MoS_2 was taken from the Materials Project database and fully optimised. The 2H structure comprises MoS_2 layers stacked along the c axis and separated by a van der Waal's gap (**Figure S17(a)**). This bulk structure was used to create a slab model consisting of 3×3 unit cells along the a and b directions and five layers stacked along the c direction with a 15 Å gap between periodic images (**Figure S17(b)**). Derivative structures were then created in which up to four of the Mo sites on the top surface were substituted with Cr, Mn, W or Re. Specifically, we considered: (1) the four possible single substitutions with each of the four alloying elements; (2) the four double substitutions with the same element; (3) the six double substitutions with two different elements; (4) the four triple substitutions with three different elements; and (5) the quadruple substitution with one of each element. In each case we optimised all symmetry-unique arrangements of the metal ions and identified the configuration with the lowest energy. The energy differences between different configurations were found to vary between 13 meV for the three configurations of the doubly-substituted MoS_2 + W, W model and 0.52 eV for the 11 configurations of the triply-substituted MoS_2 + Cr, Mn, Re model (**Table S7**). Across the five sets of models we considered a total of 130 configurations from which 19 were selected (**Figures S19(a)-S37(a)**).

For each selected configuration we calculated the electronic density of states (DoS), including the projections onto the different atomic species, in order to assess the impact of the substitutions on the electronic structure (**Figures S38-S56**). To determine whether the substitutions affect the relative positions of the valence- and conduction-band edges, we also compared DoS curves with the energies referenced to the average 1s core level of the nine Mo atoms in the central layer of the slab (**Figures S57**).

It has previously been shown that the Gibbs free energy of H adsorption at the surface, G_{H^*}, is a good proxy for HER activity.[38] G_{H^*} is calculated as[38c]:

$$G_{H^*} = \left[E_{Surf+H} - E_{Surf} - \frac{1}{2}E_{H_2}\right] + \Delta E_{ZPE} - T\Delta S_H = E_{ads} + \Delta E_{ZPE} - T\Delta S_H$$

Here E_{Surf}, E_{Surf+H} and E_{H_2} are the energies of the pristine surface, the surface with a bound H atom and a H_2 molecule, respectively, and are used to calculate the adsorption energy E_{ads} of H to the surface. ΔE_{ZPE} and ΔS_H are respectively the (positive) change in zero-point vibrational energy and (negative) change in entropy of H on binding. For binding on the Cu(111) surface ΔE_{ZPE} is calculated to be 40 meV, while ΔS_H can be approximated as minus half the standard entropy of H_2, $S^0_{H_2}/2$, to give 0.2 eV.[38c] If G_{H^*} > 0, the binding of H at the surface is energetically disfavoured, resulting in slow H adsorption. On the other hand, if G_{H^*} < 0 the binding is energetically favoured, resulting in slow H release. An optimal catalyst therefore requires thermodynamically-neutral H binding with $G_{H^*} \approx 0$, for which E_{ads} should be around -0.24 eV to offset the ΔE_{ZPE} and $-T\Delta S_H$ terms.

We calculated the H binding energies for our pristine MoS_2 surface and the 19 substituted surfaces (**Figure S18(a)**, **Figures S19(a)-S37(a)**). For each model, a H atom was placed at an initial distance of 2 Å above each of the symmetry-unique surface sites and the resulting models optimised. In total, we considered 266 initial configurations across pristine MoS_2 and the 19 substituted models. The E_{ads} were then calculated from the energies of the optimised slab models with and without adsorbed H and the energy of a reference gas-phase H_2 molecule. This procedure gives us a range of H binding energies, which are displayed in **Figure 5** in the main text and summarised in **Table S7**. The optimised models of the MoS_2 slab with H atoms adsorbed at the two unique surface sites are shown in **Figures S17(b) and S18(c)**, and the H adsorption configurations with the lowest energies for each of the substituted surfaces are shown in **Figures S19(b)-S37(b)**. We note that the positions of the H atoms were not constrained during the optimisations, so it was possible for H atoms placed initially above one surface site to move to a different (e.g. neighbouring) site during the structural relaxation.

All modelling was performed using pseudopotential plane-wave density-functional theory (DFT), as implemented in the Vienna *Ab initio* Simulation Package (VASP) code.[39]

Electron exchange and correlation were modelled using the PBE generalised-gradient approximation (GGA) exchange-correlation functional[40] with the DFT+U correction applied

to the transition-metal d orbitals according to the method of Dudarev *et al.* (i.e. PBE+U).[41] The U values were taken from the Materials Project (MP)[42] and are calibrated against redox energies according to the method in Ref. [43]: Cr - 3.7 eV, Mn - 3.9 eV, Mo - 4.38 eV, and W - 6.2 eV. The MP does not list a U value for Re. Extensive testing on the singly-substituted MoS_2 + Re slab model found that DFT+U calculations with a correction of U = 1-8 eV applied to the Re d orbitals were numerically unstable, and we were therefore forced to disable the correction for this element.

All calculations were performed using spin polarisation. Calculations on MoS_2 predicted a non-magnetic electronic ground state. The slabs were therefore started with an initial magnetic moment of M = 0 on the Mo and S atoms. For Cr and W we assumed the same electronic configuration as Mo and set an initial magnetic moment of M = 0 on these atoms. For Mn and Re, test calculations on the singly-substituted slabs with initial moments of M = 0, 1 and 3 BM yielded moments of M = 1 and M = 0.5 BM on Mn and Re, respectively, after geometry optimisation, so these were set as the initial moments on these atoms in all other calculations. For the H-adsorption calculations we set an initial moment of M = 1 BM on the H atom. For simplicity, we set an initial ferromagnetic ordering in calculations on models containing multiple atoms with non-zero initial moments. We note that we did not constrain the magnetic moments, and in some models the initial moments relaxed to different values during the geometry optimisations.

Electronic-structure calculations were performed on the bare slab models using the r^2SCAN meta-GGA functional,[44] which we previously found to give better bandgaps than GGA+U at a more manageable computational cost than a hybrid functional such as HSE06.[45]

The ion cores were modelled using projector augmented-wave (PAW) pseudopotentials[46] with the following electrons included in the valence shells: H - $1s^1$, S - $3s^2 3p^4$, Cr - $3p^6 4s^1 3d^5$, Mn - $3p^6 4s^2 3d^5$, Mo - $4p^6 5s^1 4d^5$, W - $5p^6 6s^2 5d^4$, Re - $5p^6 6s^2 5d^5$. These pseudopotentials correspond to those recommended by the Materials Project.[42]

For bulk MoS_2, the Kohn-Sham wavefunctions were expanded in a plane-wave basis with a kinetic-energy cutoff of 500 eV and the electronic Brillouin zone was integrated using a Γ-centered Monkhorst-Pack k-point mesh[47] with 10 × 10 × 2 subdivisions. These parameters were determined based on explicit convergence testing to converge the absolute

total energy to < 1 meV atom^{-1} and the cell pressure to < 1 kbar (0.1 GPa). A full geometry optimisation was performed to tolerances of 10^{-8} eV on the electronic energies and 10^{-2} eV Å^{-1} on the ionic forces. The PAW projection was performed in reciprocal space, the precision of the charge-density grids was set automatically to avoid aliasing errors, and non-spherical contributions to the gradient correction were accounted for inside the PAW spheres.

The slab models were generated from bulk MoS_2 using the Transformer code. For these models we used the same cutoff but a slightly reduced k-point sampling of $2 \times 2 \times 1$ as opposed to the $4 \times 4 \times 1$ or $3 \times 3 \times 1$ meshes that should be used based on the k-point mesh used for bulk MoS_2 and the three-fold expansion in the a and b directions. The optimisations were performed at fixed volume with reduced tolerances of 10^{-6} eV on the electronic energy and 10^{-3} eV on the total energy and using real-space PAW projection. These reduced settings were tested against parameters equivalent to those used for bulk MoS_2 and found to give H adsorption energies on MoS_2 within 5 meV of the more accurate values. For the slab models a dipole correction was also applied along the z direction normal to the surface.

Finally, to determine a reference energy for H_2 a single molecule was placed at the centre of a cubic box with, initially, at least 15 Å between the closest atoms in adjacent images and optimised at constant volume using the same 500 eV cutoff as for the other models, Γ-point k-point sampling, and the more accurate convergence settings used for bulk MoS_2.

Table S7. Summary of the surface-slab models examined in this study. For each model the table lists the number of symmetry-independent metal substitutions n_{M}, the difference in energy between the highest- and lowest-energy configurations ΔE_{M}, the number of symmetry-independent H adsorption sites n_{H} in the lowest-energy configuration of the substituted metals, and the minimum and maximum H adsorption energies E_{ads} across these sites.

				E_{ads} [eV]	
	n_{M}	ΔE_{M} [eV]	n_{H}	Min	Max
MoS_2	-	-	2	1.608	2.116
+ Cr	1	-	7	0.368	1.812
+ Mn	1	-	7	-0.063	1.676
+ W	1	-	7	1.160	2.184
+ Re	1	-	7	1.197	2.190
+ Cr, Cr	2	0.222	12	0.435	1.546
+ Mn, Mn	2	0.363	12	-0.221	1.058
+ W, W	2	0.013	7	1.393	2.224
+ Re, Re	2	0.068	7	1.261	2.206
+ Cr, Mn	3	0.213	18	-0.178	1.348
+ Cr, W	3	0.044	18	0.352	1.423
+ Cr, Re	3	0.295	18	0.240	1.723
+ Mn, W	3	0.095	18	-0.010	1.161
+ Mn, Re	3	0.326	18	-0.077	1.323
+ W, Re	3	0.022	18	0.925	1.795
+ Cr, Mn, W	11	0.284	18	-0.176	1.665
+ Cr, Mn, Re	11	0.523	18	-0.212	1.119
+ Cr, W, Re	11	0.371	18	0.234	2.085
+ Mn, W, Re	11	0.400	18	0.050	1.551
+ Cr, Mn, W, Re	56	0.461	18	-0.032	1.553

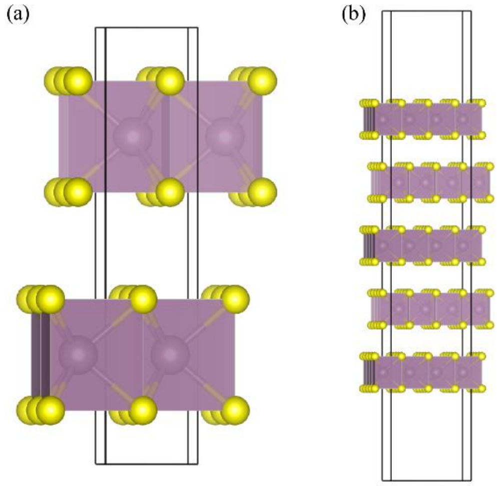

Figure S17. Optimised structure of bulk 2H MoS_2 (a) and the optimised five-layer (001) surface slab model (b). The Mo and S atoms are shown in light purple and yellow, respectively, and the MoS_2 polyhedra are shown as transparent overlays. These images were prepared using the VESTA software.[48]

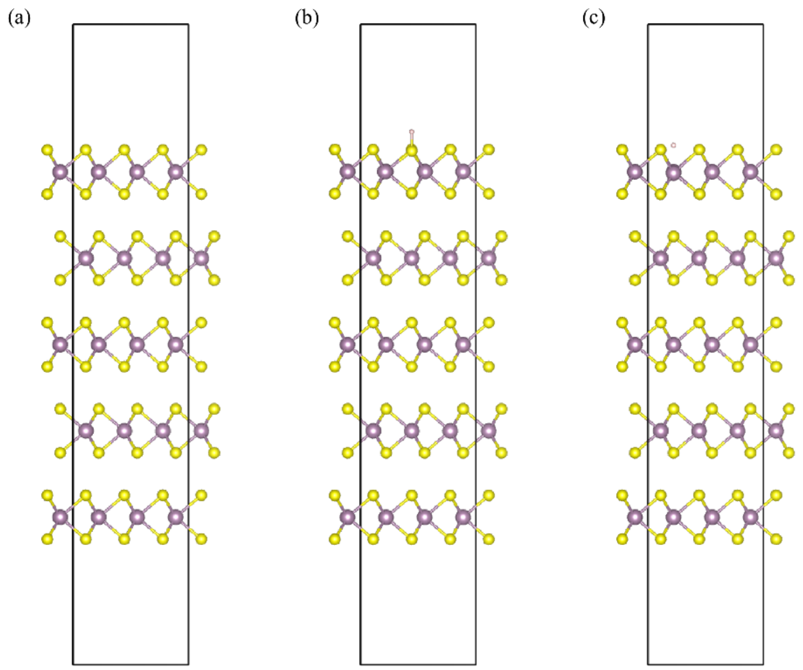

Figure S18. Optimised pristine MoS_2 surface slab (a) and optimised slabs with H atoms adsorbed at the two unique surface sites (b/c). The atoms are coloured as follows: Mo - light purple, S - yellow, H - white. These images were prepared using the VESTA software.[48]

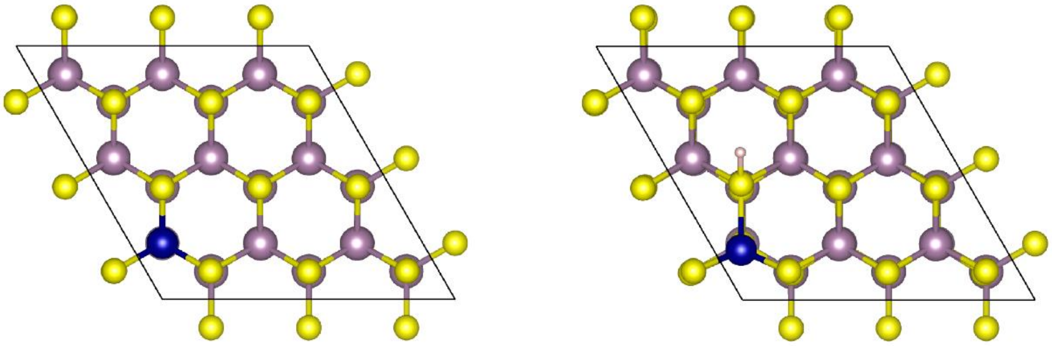

(a) (b)

Figure S19. Optimised MoS_2 + Cr surface slab (a) and lowest-energy MoS_2 + Cr slab with adsorbed H (b). Atom colours: Mo - light purple, S - yellow, Cr - blue, H - white. These images were prepared using VESTA.[48]

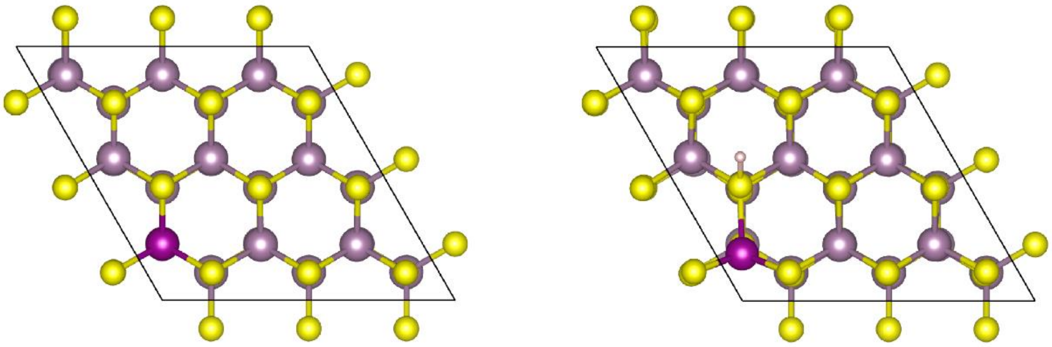

(a) (b)

Figure S20. Optimised MoS_2 + Mn surface slab (a) and lowest-energy MoS_2 + Mn slab with adsorbed H (b). Atom colours: Mo - light purple, S - yellow, Mn - dark purple, H - white. These images were prepared using VESTA.[48]

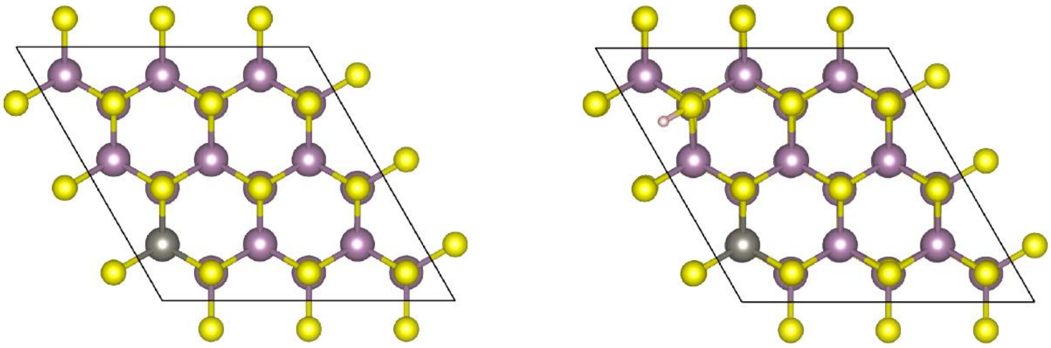

(a) (b)

Figure S21. Optimised MoS_2 + W surface slab (a) and lowest-energy MoS_2 + W slab with adsorbed H (b). Atom colours: Mo - light purple, S - yellow, W - dark grey, H - white. These images were prepared using VESTA.[48]

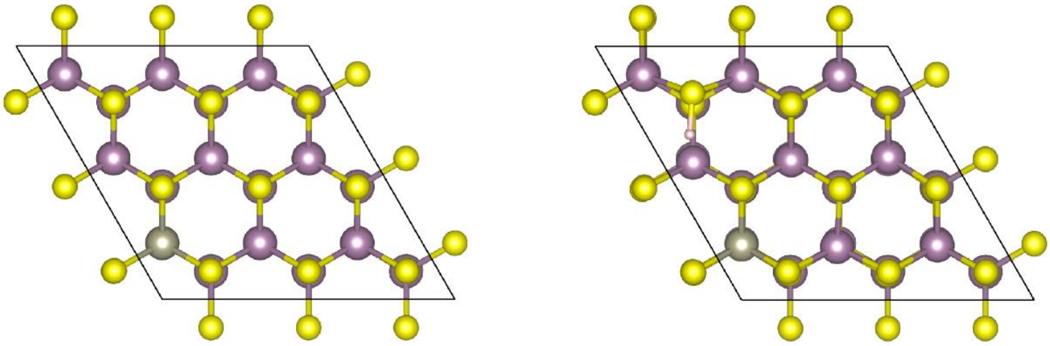

(a) (b)

Figure S22. Optimised MoS_2 + Re surface slab (a) and lowest-energy MoS_2 + Re slab with adsorbed H (b). Atom colours: Mo - light purple, S - yellow, Re - light grey, H - white. These images were prepared using VESTA.[48]

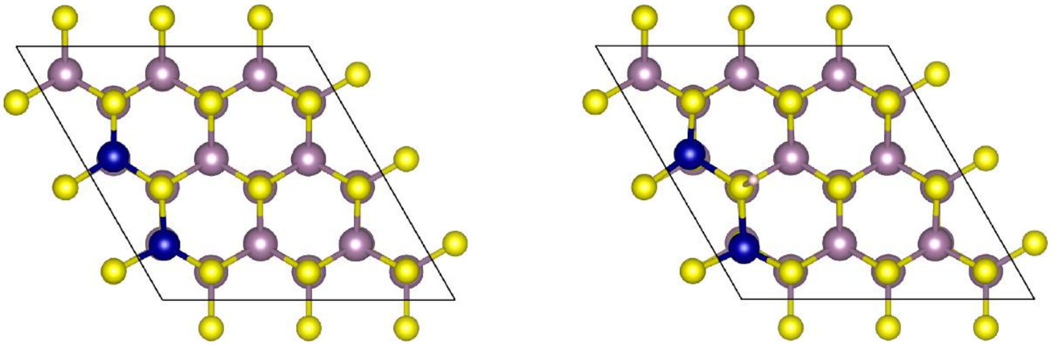

(a) (b)

Figure S23. Lowest-energy optimised MoS_2 + Cr, Cr surface slab (a) and lowest-energy MoS_2 + Cr, Cr slab with adsorbed H (b). Atom colours: Mo - light purple, S - yellow, Cr - blue, H - white. These images were prepared using VESTA.[48]

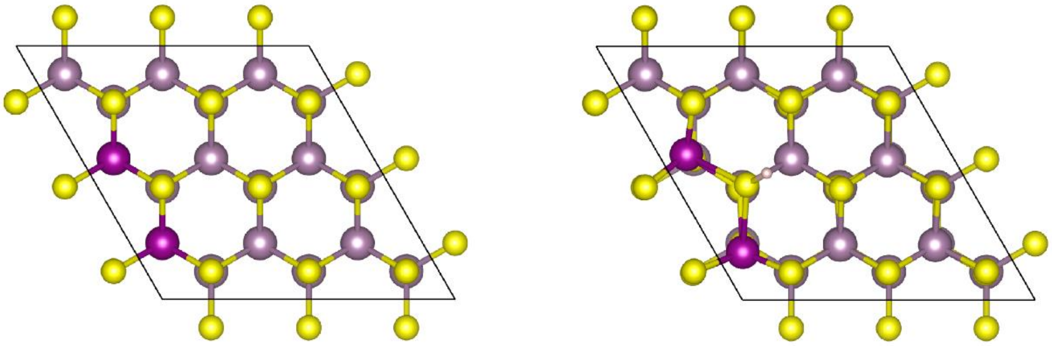

(a) (b)

Figure S24. Lowest-energy optimised MoS_2 + Mn, Mn surface slab (a) and lowest-energy MoS_2 + Mn, Mn slab with adsorbed H (b). Atom colours: Mo - light purple, S - yellow, Mn - dark purple, H - white. These images were prepared using VESTA.[48]

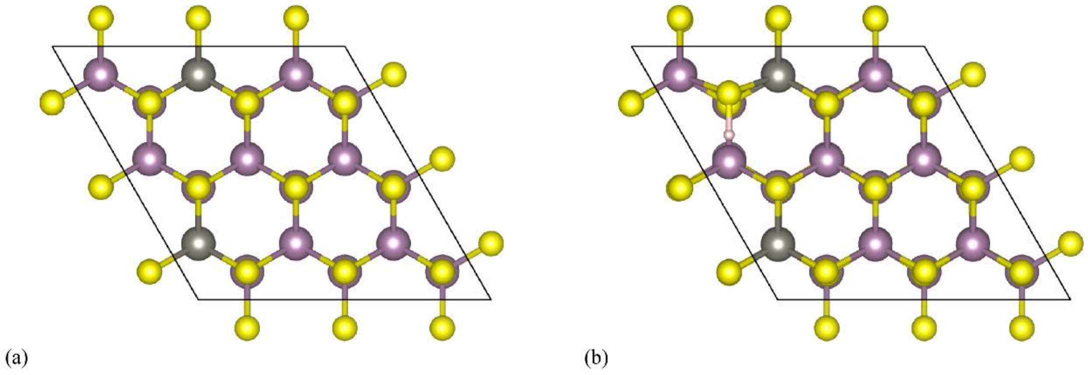

Figure S25. Lowest-energy optimised MoS_2 + W, W surface slab (a) and lowest-energy MoS_2 + W, W slab with adsorbed H (b). Atom colours: Mo - light purple, S - yellow, W - dark grey, H - white. These images were prepared using VESTA.[48]

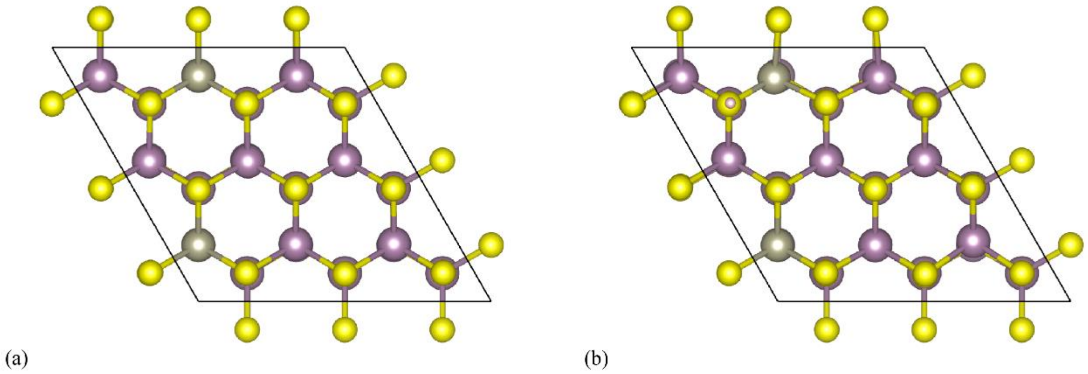

Figure S26. Lowest-energy optimised MoS_2 + Re, Re surface slab (a) and lowest-energy MoS_2 + Re, Re slab with adsorbed H (b). Atom colours: Mo - light purple, S - yellow, Re - light grey, H - white. These images were prepared using VESTA.[48]

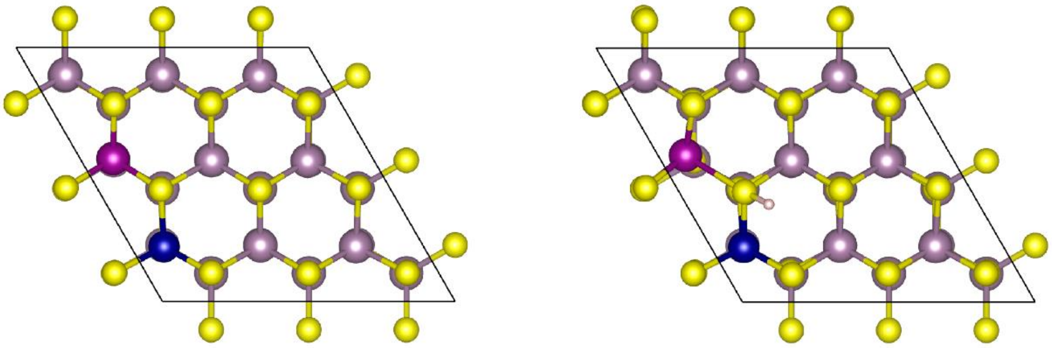

(a) (b)

Figure S27. Lowest-energy optimised MoS_2 + Cr, Mn surface slab (a) and lowest-energy MoS_2 + Cr, Mn slab with adsorbed H (b). Atom colours: Mo - light purple, S - yellow, Cr - blue, Mn - dark purple, H - white. These images were prepared using VESTA.[48]

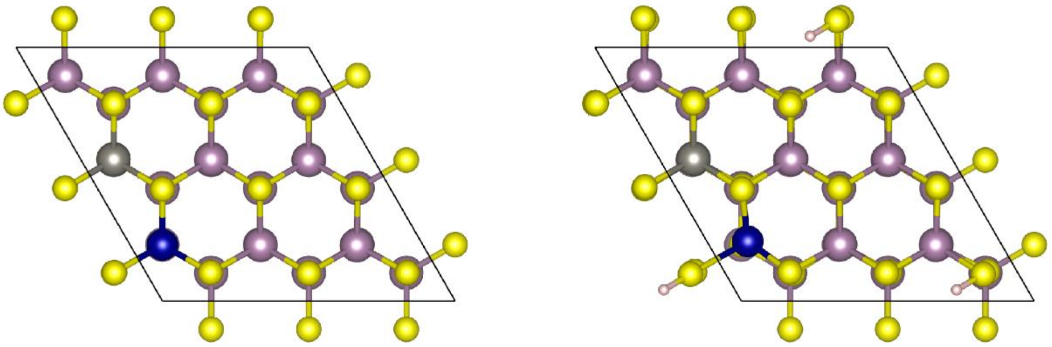

(a) (b)

Figure S28. Lowest-energy optimised MoS_2 + Cr, W surface slab (a) and lowest-energy MoS_2 + Cr, W slab with adsorbed H (b). Atom colours: Mo - light purple, S - yellow, Cr - blue, W - dark grey, H - white. These images were prepared using VESTA.[48]

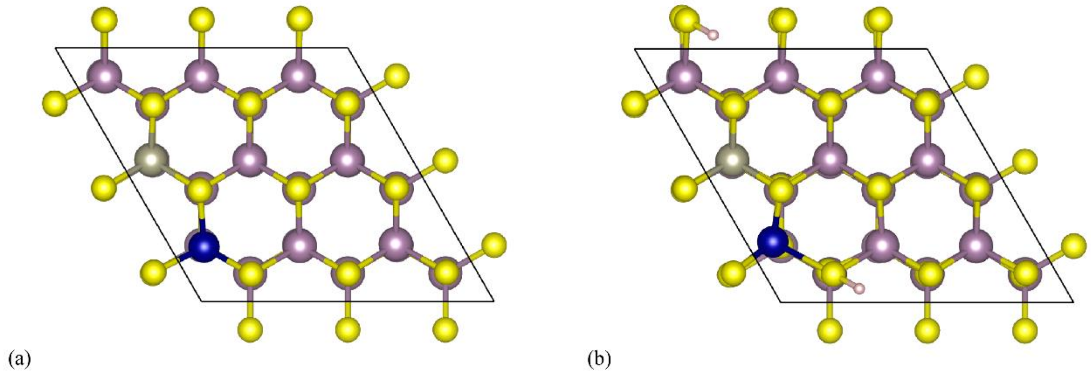

Figure S29. Lowest-energy optimised MoS_2 + Cr, Re surface slab (a) and lowest-energy MoS_2 + Cr, Re slab with adsorbed H (b). Atom colours: Mo - light purple, S - yellow, Cr - blue, Re - light grey, H - white. These images were prepared using VESTA.[48]

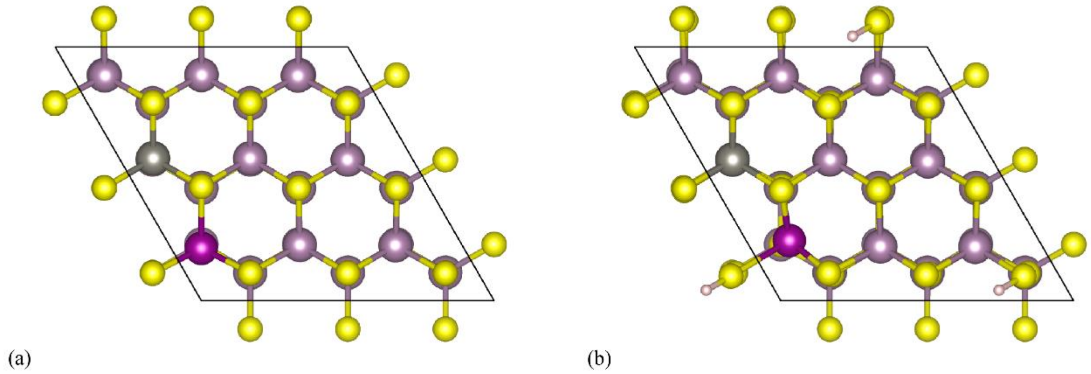

Figure S30. Lowest-energy optimised MoS_2 + Mn, W surface slab (a) and lowest-energy MoS_2 + Mn, W slab with adsorbed H (b). Atom colours: Mo - light purple, S - yellow, Mn - dark purple, W - dark grey, H - white. These images were prepared using VESTA.[48]

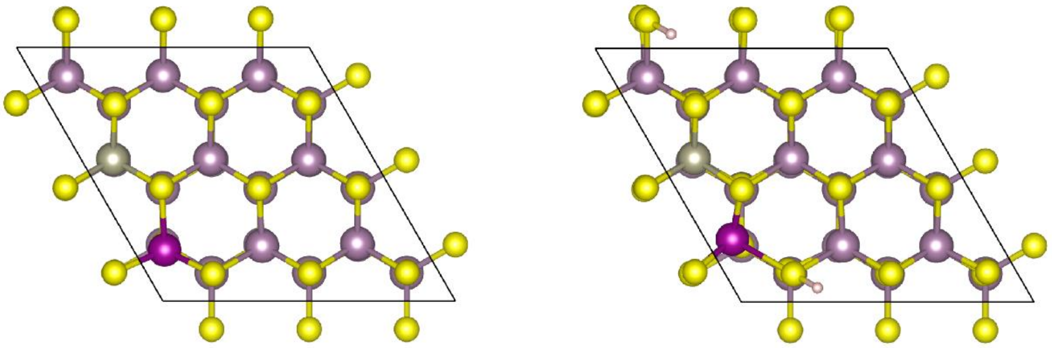

(a) (b)

Figure S31. Lowest-energy optimised MoS_2 + Mn, Re surface slab (a) and lowest-energy MoS_2 + Mn, Re slab with adsorbed H (b). Atom colours: Mo - light purple, S - yellow, Mn - dark purple, Re - light grey, H - white. These images were prepared using VESTA.[48]

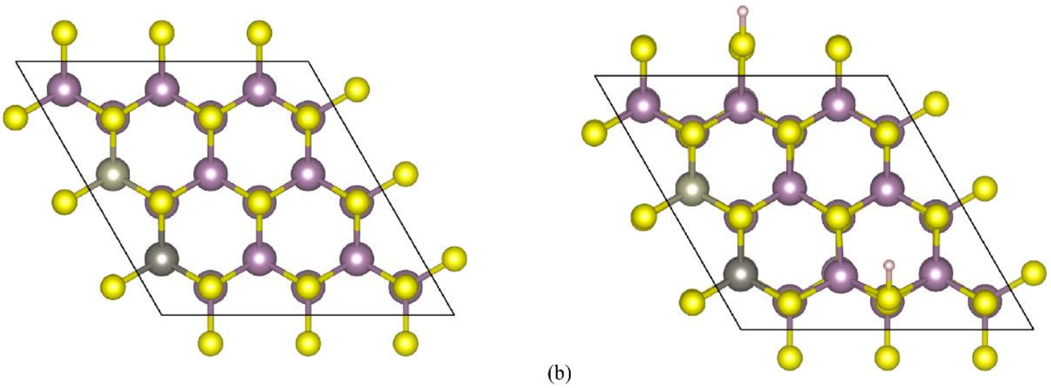

(a) (b)

Figure S32. Lowest-energy optimised MoS_2 + W, Re surface slab (a) and lowest-energy MoS_2 + W, Re slab with adsorbed H (b). Atom colours: Mo - light purple, S - yellow, W - dark grey, Re - light grey, H - white. These images were prepared using VESTA.[48]

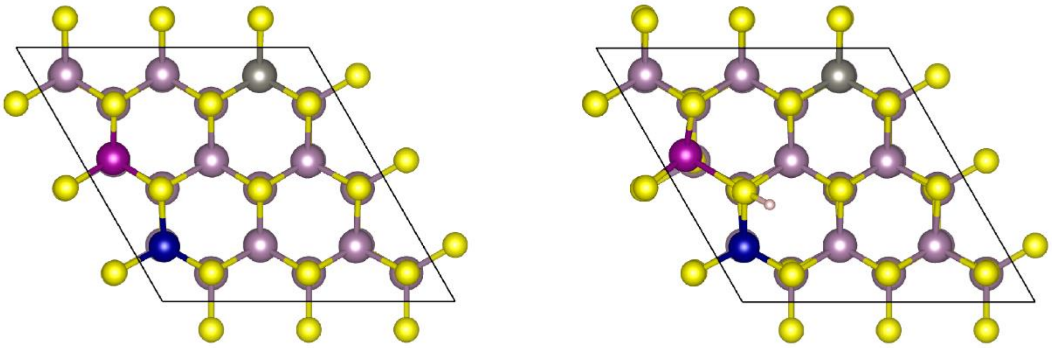

(a) (b)

Figure S33. Lowest-energy optimised MoS_2 + Cr, Mn, W surface slab (a) and lowest-energy MoS_2 + Cr, Mn W slab with adsorbed H (b). Atom colours: Mo - light purple, S - yellow, Cr - blue, Mn - dark purple, W - dark grey, H - white. These images were prepared using VESTA.[48]

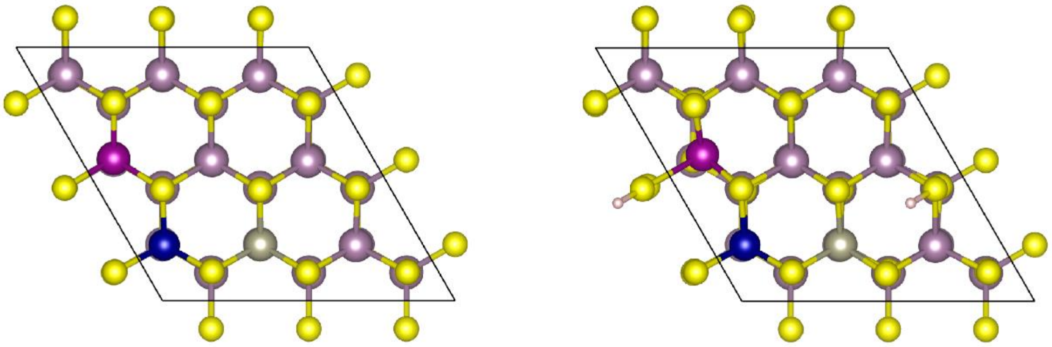

(a) (b)

Figure S34. Lowest-energy optimised MoS_2 + Cr, Mn, Re surface slab (a) and lowest-energy MoS_2 + Cr, Mn, Re slab with adsorbed H (b). Atom colours: Mo - light purple, S - yellow, Cr - blue, Mn - dark purple, Re - light grey, H - white. These images were prepared using VESTA.[48]

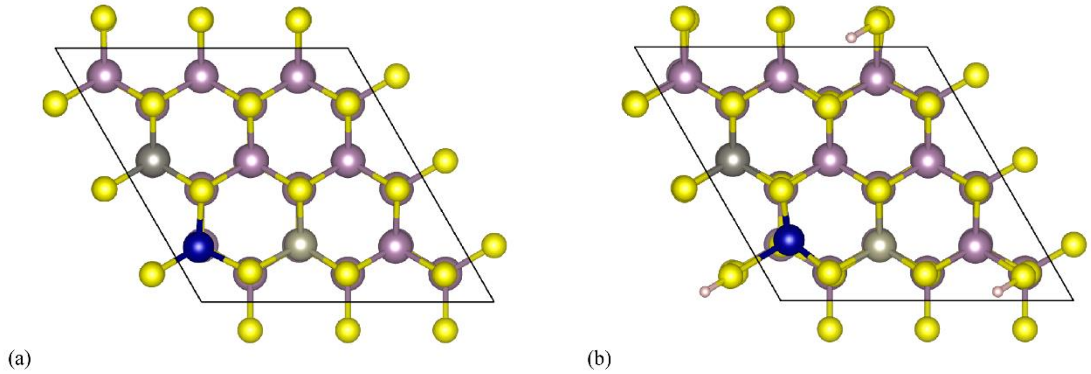

Figure S35. Lowest-energy optimised MoS_2 + Cr, W, Re surface slab (a) and lowest-energy MoS_2 + Cr, W, Re slab with adsorbed H (b). Atom colours: Mo - light purple, S - yellow, Cr - blue, W - dark grey, Re - light grey, H - white. These images were prepared using VESTA.[48]

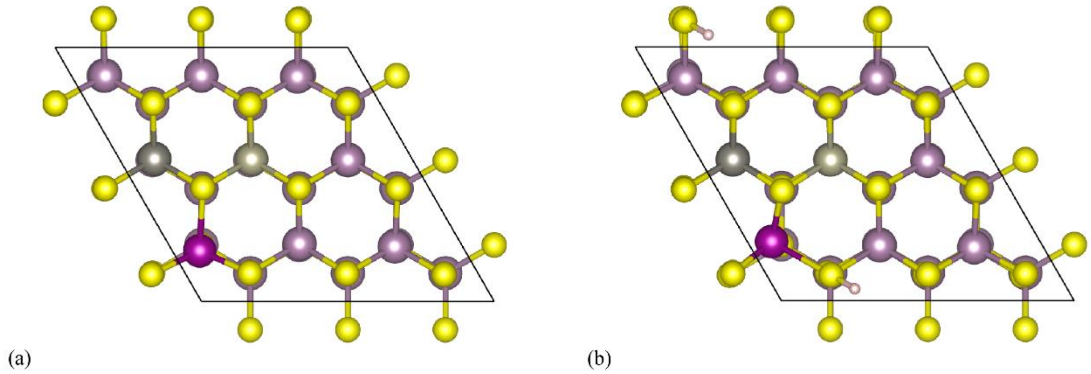

Figure S36. Lowest-energy optimised MoS_2 + Mn, W, Re surface slab (a) and lowest-energy MoS_2 + Mn, W, Re slab with adsorbed H (b). Atom colours: Mo - light purple, S - yellow, Mn - dark purple, W - dark grey, Re - light grey, H - white. These images were prepared using VESTA.[48]

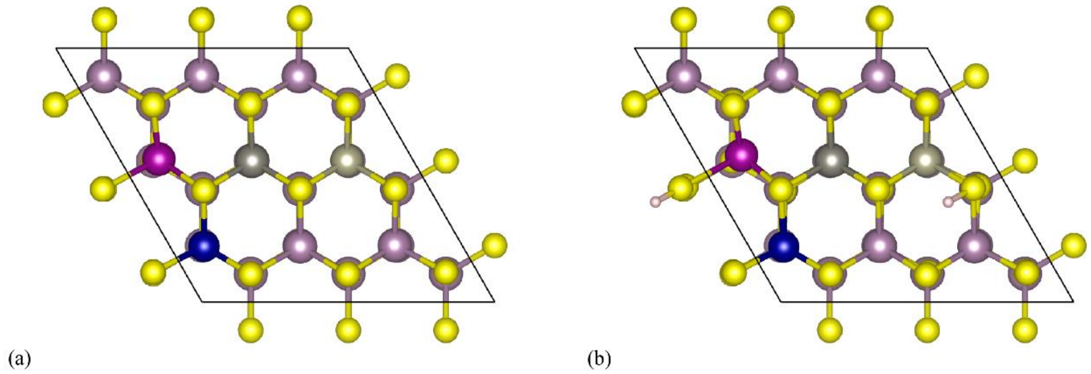

Figure S37. Lowest-energy optimised MoS_2 + Cr, Mn, W, Re surface slab (a) and lowest-energy MoS_2 + Cr, Mn, W, Re slab with adsorbed H (b). Atom colours: Mo - light purple, S - yellow, Cr - blue, Mn - dark purple, W - dark grey, Re - light grey, H - white. These images were prepared using VESTA.[48]

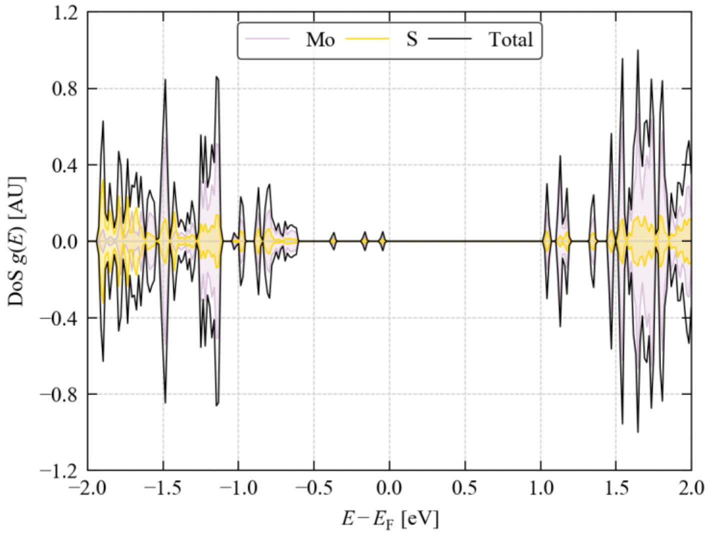

Figure S38. Electronic density of states $g(E)$ (DoS) of the optimised pristine MoS_2 surface slab in the vicinity of the Fermi energy E_F. The projections of the DoS onto atomic species are shown as a stacked area plot and coloured as follows: Mo - light purple, S - yellow. The two spin components are shown as positive and negative values.

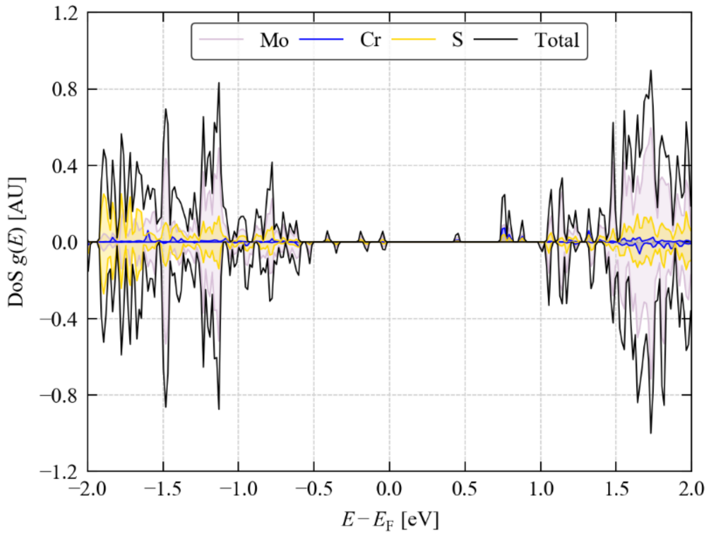

Figure S39. Electronic density of states $g(E)$ (DoS) of the optimised MoS_2 + Cr surface slab in the vicinity of the Fermi energy E_F. The projections of the DoS onto atomic species are shown as a stacked area plot and coloured as follows: Mo - light purple, S - yellow, Cr - blue. The two spin components are shown as positive and negative values.

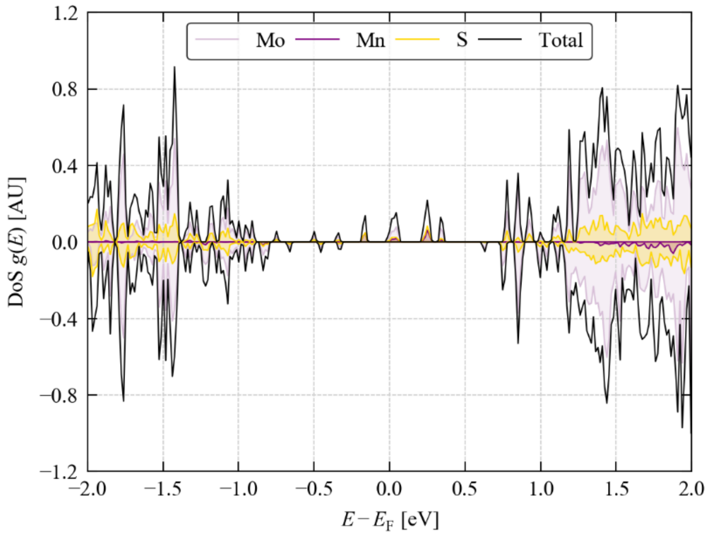

Figure S40. Electronic density of states $g(E)$ (DoS) of the optimised MoS_2 + Mn surface slab in the vicinity of the Fermi energy E_F. The projections of the DoS onto atomic species are shown as a stacked area plot and coloured as follows: Mo - light purple, S - yellow, Mn - dark purple. The two spin components are shown as positive and negative values.

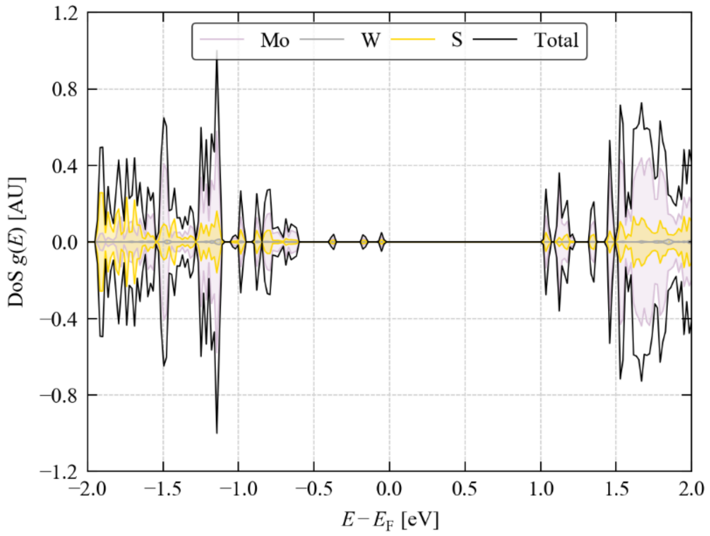

Figure S41. Electronic density of states $g(E)$ (DoS) of the optimised MoS_2 + W surface slab in the vicinity of the Fermi energy E_F. The projections of the DoS onto atomic species are shown as a stacked area plot and coloured as follows: Mo - light purple, S - yellow, W - dark grey. The two spin components are shown as positive and negative values.

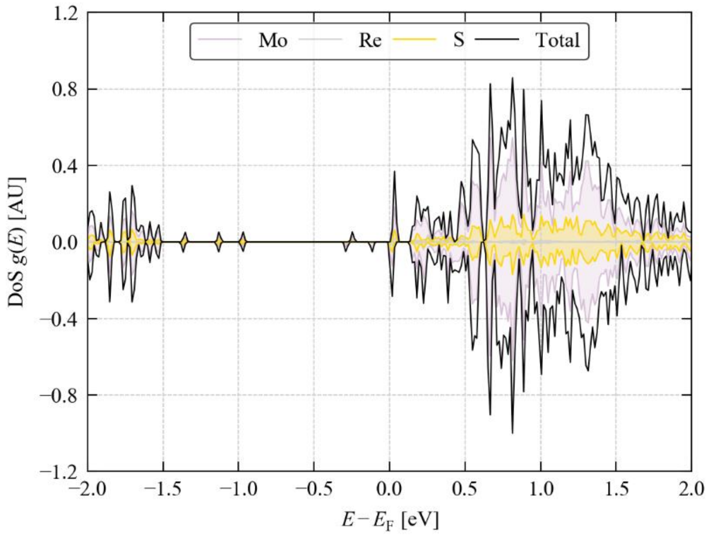

Figure S42. Electronic density of states $g(E)$ (DoS) of the optimised MoS_2 + Re surface slab in the vicinity of the Fermi energy E_F. The projections of the DoS onto atomic species are shown as a stacked area plot and coloured as follows: Mo - light purple, S - yellow, Re - light grey. The two spin components are shown as positive and negative values.

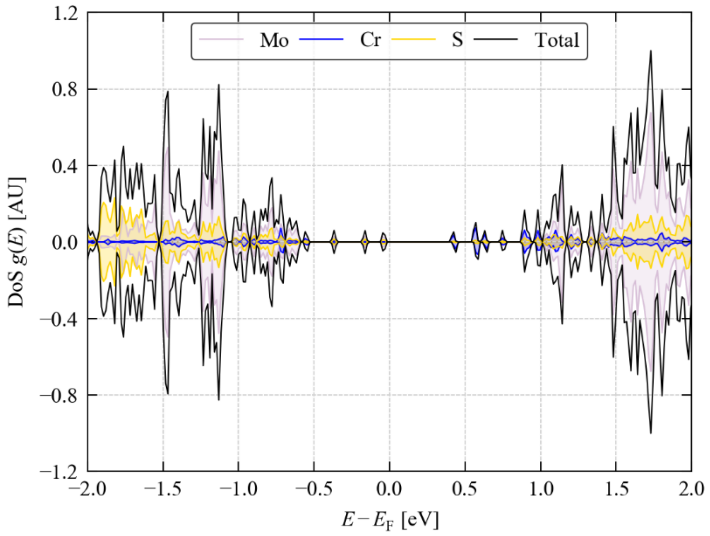

Figure S43. Electronic density of states $g(E)$ (DoS) of the optimised MoS_2 + Cr, Cr surface slab in the vicinity of the Fermi energy E_F. The projections of the DoS onto atomic species are shown as a stacked area plot and coloured as follows: Mo - light purple, S - yellow, Cr - blue. The two spin components are shown as positive and negative values.

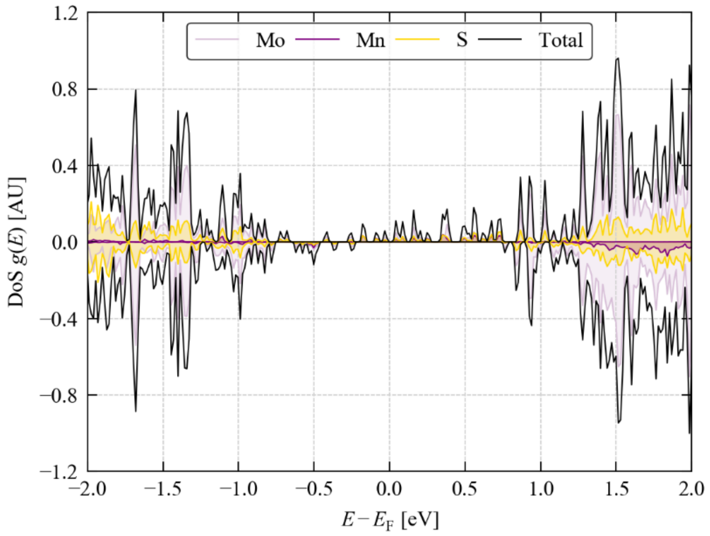

Figure S44. Electronic density of states $g(E)$ (DoS) of the optimised MoS_2 + Mn, Mn surface slab in the vicinity of the Fermi energy E_F. The projections of the DoS onto atomic species are shown as a stacked area plot and coloured as follows: Mo - light purple, S - yellow, Mn - dark purple. The two spin components are shown as positive and negative values.

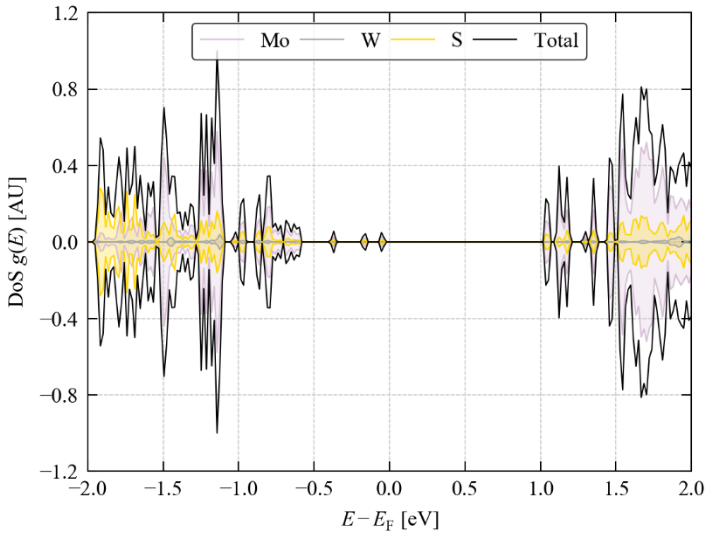

Figure S45. Electronic density of states $g(E)$ (DoS) of the optimised MoS_2 + W, W surface slab in the vicinity of the Fermi energy E_F. The projections of the DoS onto atomic species are shown as a stacked area plot and coloured as follows: Mo - light purple, S - yellow, W - dark grey. The two spin components are shown as positive and negative values.

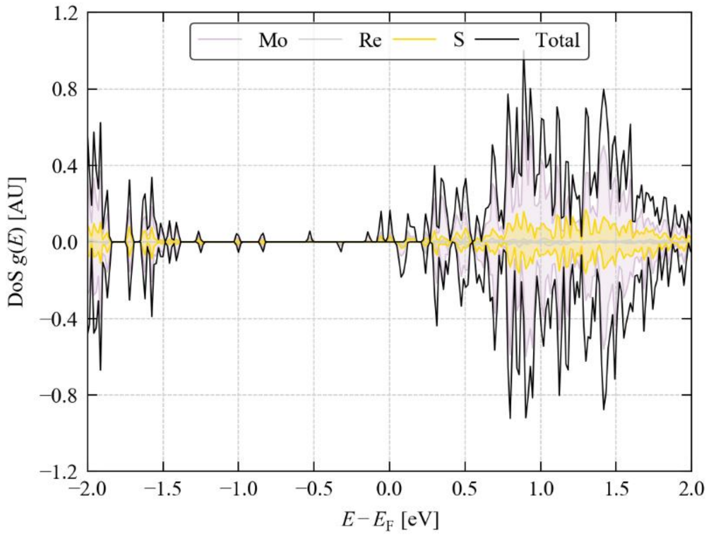

Figure S46. Electronic density of states $g(E)$ (DoS) of the optimised MoS_2 + Re, Re surface slab in the vicinity of the Fermi energy E_F. The projections of the DoS onto atomic species are shown as a stacked area plot and coloured as follows: Mo - light purple, S - yellow, Re - light grey. The two spin components are shown as positive and negative values.

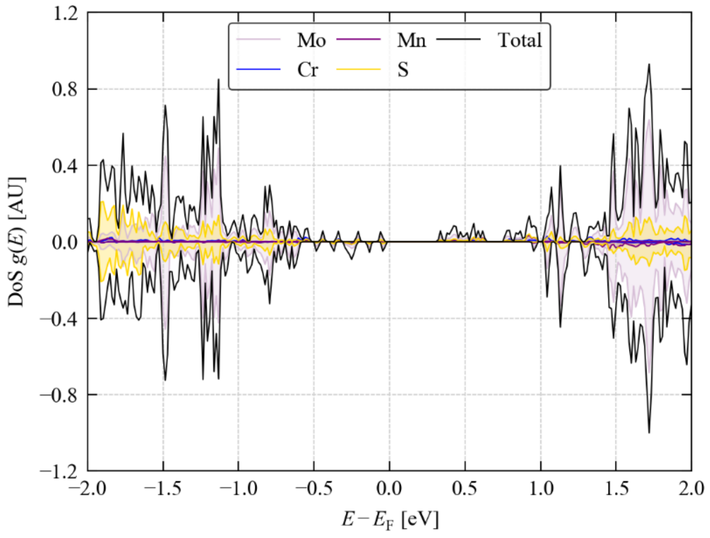

Figure S47. Electronic density of states $g(E)$ (DoS) of the optimised MoS_2 + Cr, Mn surface slab in the vicinity of the Fermi energy E_F. The projections of the DoS onto atomic species are shown as a stacked area plot and coloured as follows: Mo - light purple, S - yellow, Cr - blue, Mn - dark purple. The two spin components are shown as positive and negative values.

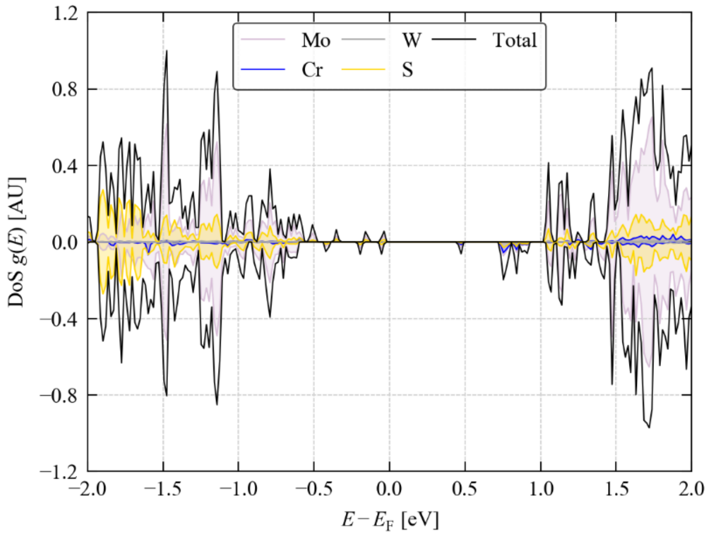

Figure S48. Electronic density of states $g(E)$ (DoS) of the optimised MoS_2 + Cr, W surface slab in the vicinity of the Fermi energy E_F. The projections of the DoS onto atomic species are shown as a stacked area plot and coloured as follows: Mo - light purple, S - yellow, Cr - blue, W - dark grey. The two spin components are shown as positive and negative values.

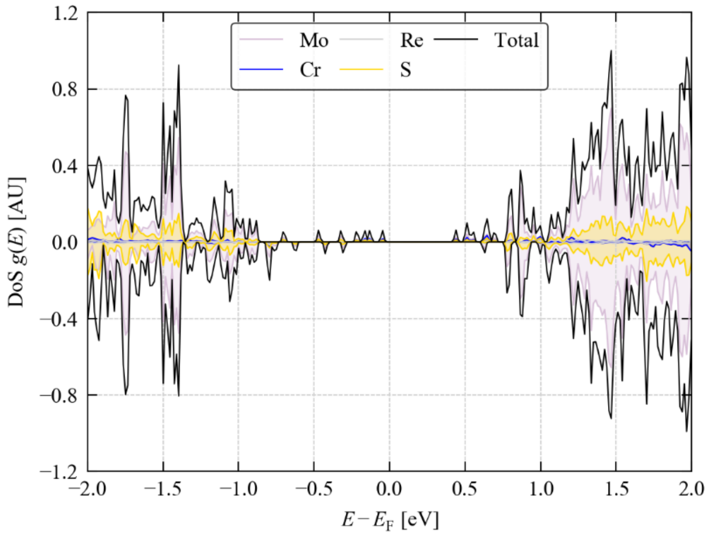

Figure S49. Electronic density of states $g(E)$ (DoS) of the optimised MoS_2 + Cr, Re surface slab in the vicinity of the Fermi energy E_F. The projections of the DoS onto atomic species are shown as a stacked area plot and coloured as follows: Mo - light purple, S - yellow, Cr - blue, Re - light grey. The two spin components are shown as positive and negative values.

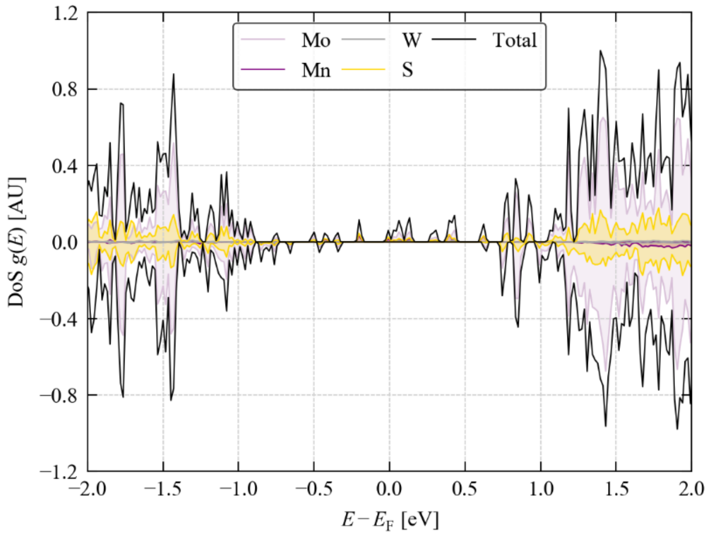

Figure S50. Electronic density of states $g(E)$ (DoS) of the optimised MoS_2 + Mn, W surface slab in the vicinity of the Fermi energy E_F. The projections of the DoS onto atomic species are shown as a stacked area plot and coloured as follows: Mo - light purple, S - yellow, Mn - dark purple, W - dark grey. The two spin components are shown as positive and negative values.

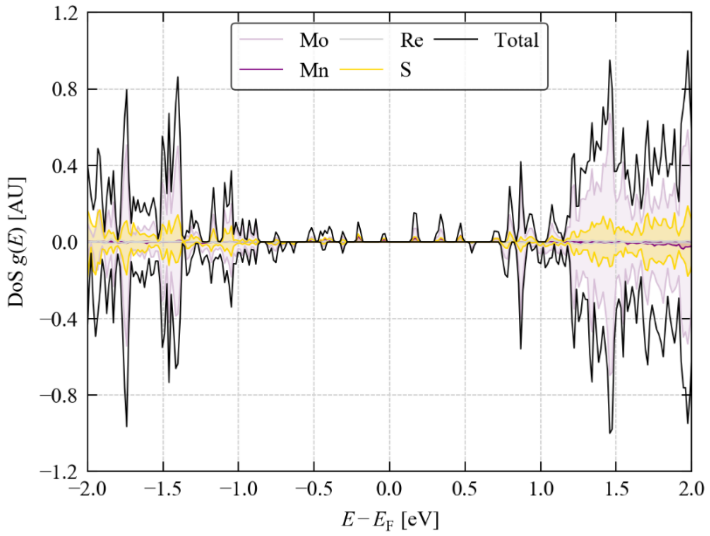

Figure S51. Electronic density of states $g(E)$ (DoS) of the optimised MoS_2 + Mn, Re surface slab in the vicinity of the Fermi energy E_F. The projections of the DoS onto atomic species are shown as a stacked area plot and coloured as follows: Mo - light purple, S - yellow, Mn - dark purple, Re - light grey. The two spin components are shown as positive and negative values.

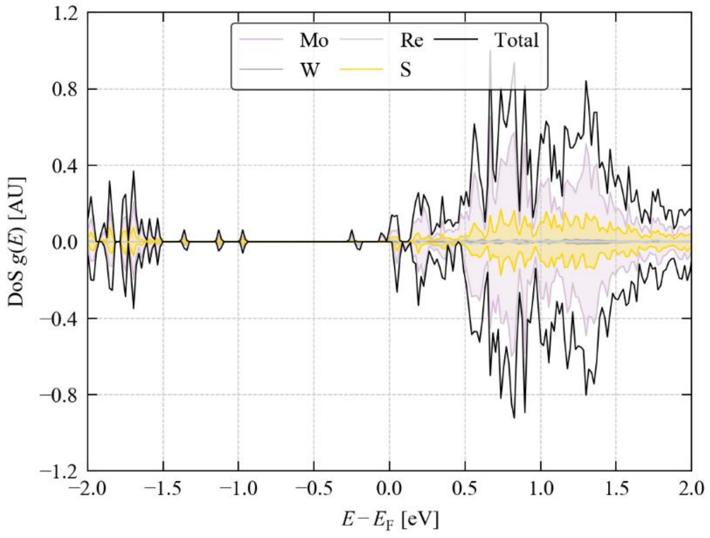

Figure S52. Electronic density of states $g(E)$ (DoS) of the optimised MoS_2 + W, Re surface slab in the vicinity of the Fermi energy E_F. The projections of the DoS onto atomic species are shown as a stacked area plot and coloured as follows: Mo - light purple, S - yellow, W - dark grey, Re - light grey. The two spin components are shown as positive and negative values.

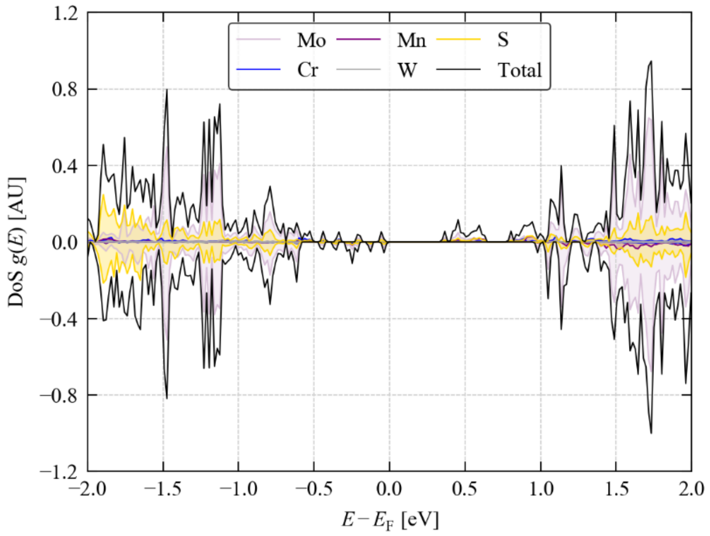

Figure S53. Electronic density of states $g(E)$ (DoS) of the optimised MoS_2 + Cr, Mn, W surface slab in the vicinity of the Fermi energy E_F. The projections of the DoS onto atomic species are shown as a stacked area plot and coloured as follows: Mo - light purple, S - yellow, Cr - blue, Mn - dark purple, W - dark grey. The two spin components are shown as positive and negative values.

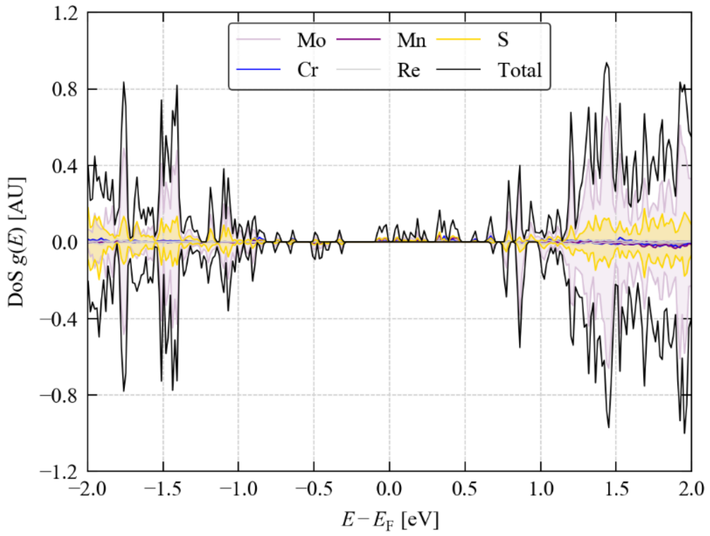

Figure S54. Electronic density of states $g(E)$ (DoS) of the optimised MoS_2 + Cr, Mn, Re surface slab in the vicinity of the Fermi energy E_F. The projections of the DoS onto atomic species are shown as a stacked area plot and coloured as follows: Mo - light purple, S - yellow, Cr - blue, Mn - dark purple, Re - light grey. The two spin components are shown as positive and negative values.

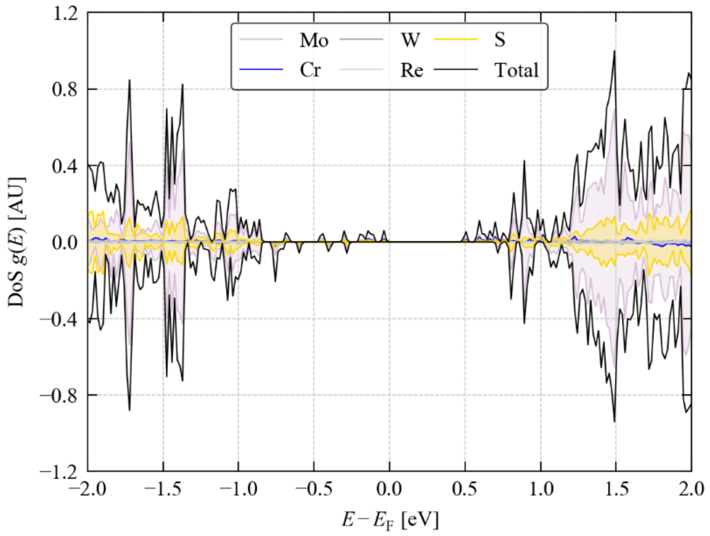

Figure S55. Electronic density of states $g(E)$ (DoS) of the optimised MoS_2 + Cr, W, Re surface slab in the vicinity of the Fermi energy E_F. The projections of the DoS onto atomic species are shown as a stacked area plot and coloured as follows: Mo - light purple, S - yellow, Cr - blue, W - dark grey, Re - light grey. The two spin components are shown as positive and negative values.

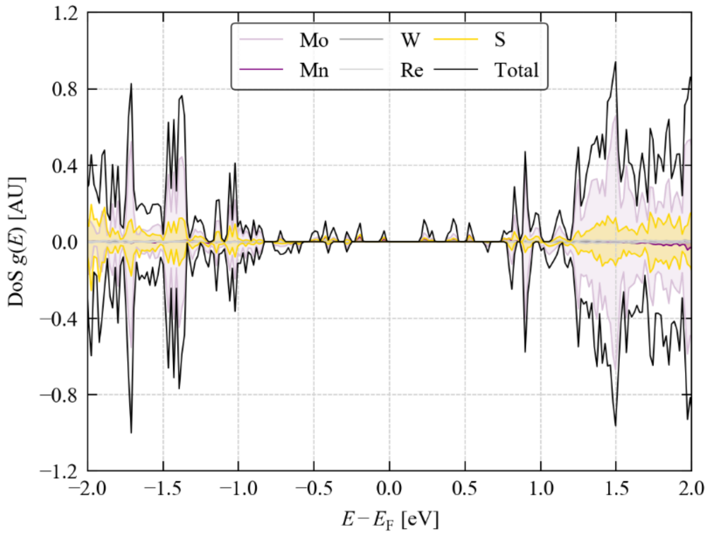

Figure S56. Electronic density of states $g(E)$ (DoS) of the optimised MoS_2 + Mn, W, Re surface slab in the vicinity of the Fermi energy E_F. The projections of the DoS onto atomic species are shown as a stacked area plot and coloured as follows: Mo - light purple, S - yellow, Mn - dark purple, W - dark grey, Re - light grey. The two spin components are shown as positive and negative values.

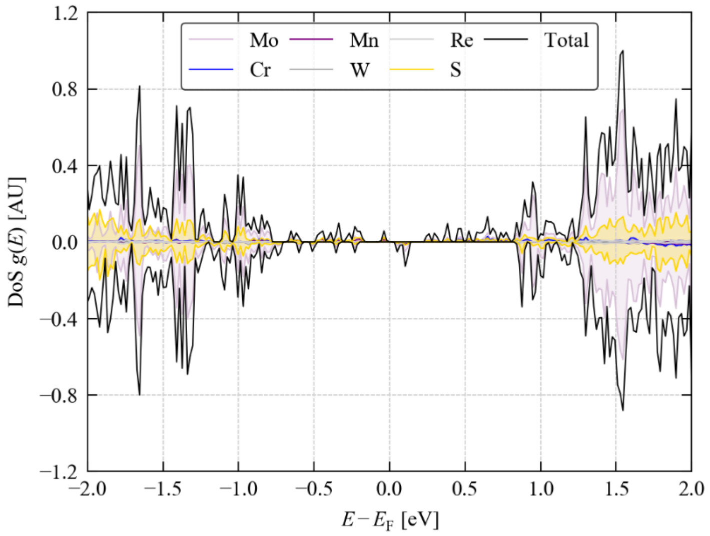

Figure S57. Electronic density of states $g(E)$ (DoS) of the optimised MoS_2 + Cr, Mn, W, Re surface slab in the vicinity of the Fermi energy E_F. The projections of the DoS onto atomic species are shown as a stacked area plot and coloured as follows: Mo - light purple, S - yellow, Cr - blue, Mn - dark purple, W - dark grey, Re - light grey. The two spin components are shown as positive and negative values.

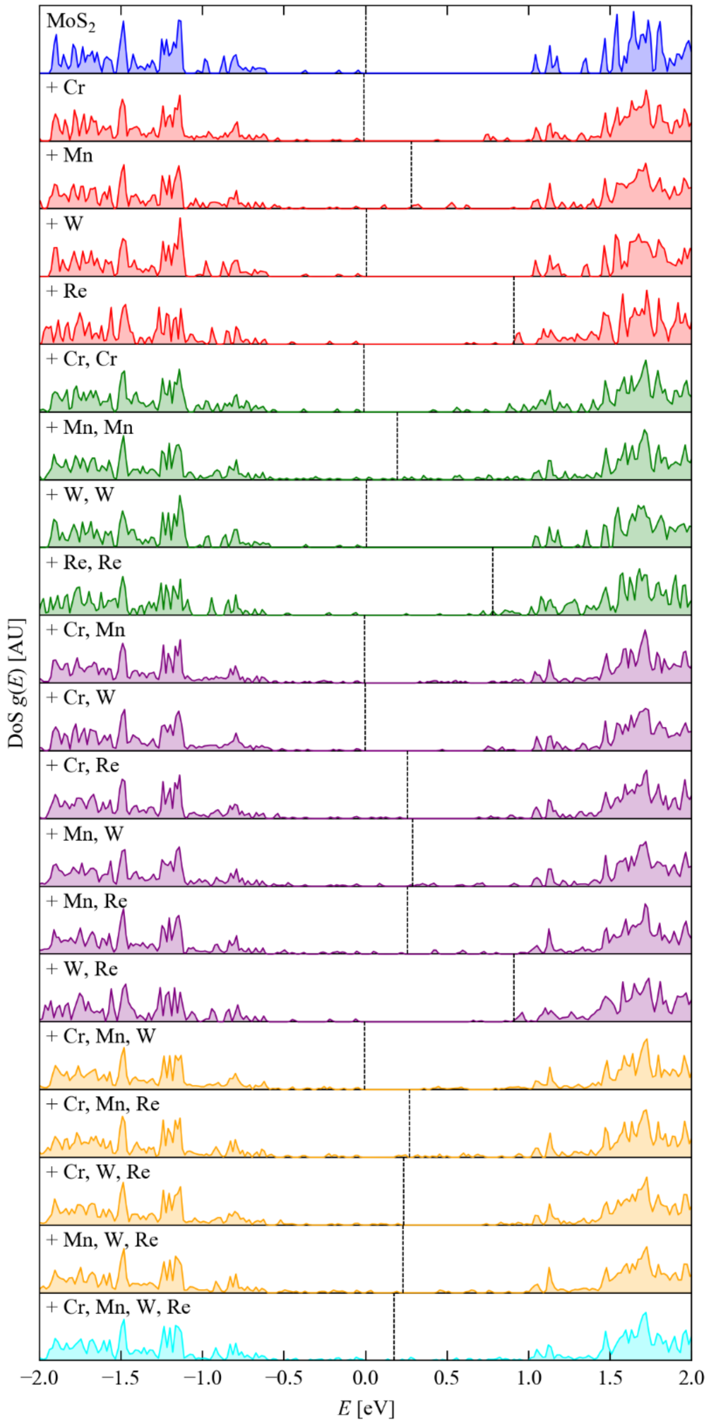

Figure S58. Comparison of the electronic density of states $g(E)$ (DoS) of the pristine MoS_2 slab and the 19 substituted slabs examined in this work. On each curve the two spin components have been summed and the Fermi energy E_F is shown by a vertical dashed line. The energies are referenced to the average 1s core level of the nine Mo atoms in the central layer of the slabs (c.f. **Figs. S10(b)/S11(a)**) and the energy zero is set to the E_F of the pristine MoS_2 slab.

References:

[1] N. Zeng, Y.-C. Wang, J. Neilson, S. M. Fairclough, Y. Zou, A. G. Thomas, R. J. Cernik, S. J. Haigh, D. J. Lewis, *Chemistry of Materials* **2020**, *32*, 7895-7907.

[2] N. Al-Dulaimi, E. A. Lewis, N. Savjani, P. D. McNaughter, S. J. Haigh, M. A. Malik, D. J. Lewis, P. O'Brien, *Journal of Materials Chemistry C* **2017**, *5*, 9044-9052.

[3] G. Murtaza, S. Alderhami, Y. T. Alharbi, U. Zulfiqar, M. Hossin, A. M. Alanazi, L. Almanqur, E. U. Onche, S. P. Venkateswaran, D. J. Lewis, *ACS Applied Energy Materials* **2020**, *3*, 1952-1961.

[4] D. J. Lewis, A. A. Tedstone, X. L. Zhong, E. A. Lewis, A. Rooney, N. Savjani, J. R. Brent, S. J. Haigh, M. G. Burke, C. A. Muryn, J. M. Raftery, C. Warrens, K. West, S. Gaemers, P. O'Brien, *Chemistry of Materials* **2015**, *27*, 1367-1374.

[5] C. R. McCormick, R. E. Schaak, *Journal of the American Chemical Society* **2021**, *143*, 1017-1023.

[6] J. Cavin, A. Ahmadiparidari, L. Majidi, A. S. Thind, S. N. Misal, A. Prajapati, Z. Hemmat, S. Rastegar, A. Beukelman, M. R. Singh, K. A. Unocic, A. Salehi-Khojin, R. Mishra, *Advanced Materials* **2021**, *33*, 2100347.

[7] R.-Z. Zhang, F. Gucci, H. Zhu, K. Chen, M. J. Reece, *Inorganic Chemistry* **2018**, *57*, 13027-13033.

[8] Z. Deng, A. Olvera, J. Casamento, J. S. Lopez, L. Williams, R. Lu, G. Shi, P. F. P. Poudeu, E. Kioupakis, *Chemistry of Materials* **2020**, *32*, 6070-6077.

[9] B. Jiang, Y. Yu, J. Cui, X. Liu, L. Xie, J. Liao, Q. Zhang, Y. Huang, S. Ning, B. Jia, B. Zhu, S. Bai, L. Chen, J. Pennycook Stephen, J. He, *Science* **2021**, *371*, 830-834.

[10] T. Ying, T. Yu, Y.-S. Shiah, C. Li, J. Li, Y. Qi, H. Hosono, *Journal of the American Chemical Society* **2021**, *143*, 7042-7049.

[11] aK.-K. Liu, W. Zhang, Y.-H. Lee, Y.-C. Lin, M.-T. Chang, C.-Y. Su, C.-S. Chang, H. Li, Y. Shi, H. Zhang, C.-S. Lai, L.-J. Li, *Nano Letters* **2012**, *12*, 1538-1544; bM. A. Baker, R. Gilmore, C. Lenardi, W. Gissler, *Applied Surface Science* **1999**, *150*, 255-262.

[12] X. Liu, X. Jiang, G. Shao, H. Xiang, Z. Li, Y. Jin, Y. Chen, H. Jiang, H. Li, J. Shui, Y. Feng, S. Liu, *Small* **2022**, *18*, 2200601.

[13] C. N. R. Rao, A. Nag, *European Journal of Inorganic Chemistry* **2010**, *2010*, 4244-4250.

[14] K. Ghosh, S. Ng, C. Iffelsberger, M. Pumera, *ACS Applied Energy Materials* **2020**, *3*, 10261-10269.

[15] Z. Cai, T. Shen, Q. Zhu, S. Feng, Q. Yu, J. Liu, L. Tang, Y. Zhao, J. Wang, B. Liu, H.-M. Cheng, *Small* **2020**, *16*, 1903181.

[16] J. Jian, H. Li, X. Sun, D. Kong, X. Zhang, L. Zhang, H. Yuan, S. Feng, *ACS Sustainable Chemistry & Engineering* **2019**, *7*, 7227-7232.

[17] M. C. Biesinger, L. W. M. Lau, A. R. Gerson, R. S. C. Smart, *Applied Surface Science* **2010**, *257*, 887-898.

[18] N. Zeng, D. G. Hopkinson, B. F. Spencer, S. G. McAdams, A. A. Tedstone, S. J. Haigh, D. J. Lewis, *Chemical Communications* **2019**, *55*, 99-102.

[19] D. Kong, H. Wang, J. J. Cha, M. Pasta, K. J. Koski, J. Yao, Y. Cui, *Nano Letters* **2013**, *13*, 1341-1347.

[20] E. P. C. Higgins, A. A. Papaderakis, C. Byrne, R. Cai, A. Elgendy, S. J. Haigh, A. S.

Walton, D. J. Lewis, R. A. W. Dryfe, *The Journal of Physical Chemistry C* **2021**, *125*, 20940-20951.

[21] Y. Shi, J. Wang, C. Wang, T.-T. Zhai, W.-J. Bao, J.-J. Xu, X.-H. Xia, H.-Y. Chen, *Journal of the American Chemical Society* **2015**, *137*, 7365-7370.

[22] E. P. C. Higgins, A. A. Papaderakis, C. Byrne, A. S. Walton, D. J. Lewis, R. A. W. Dryfe, *Electrochimica Acta* **2021**, *382*, 138257.

[23] X. Xiao, Y. Wang, X. Xu, T. Yang, D. Zhang, *Molecular Catalysis* **2020**, *487*, 110890.

[24] X. Xu, L. Liu, *Nanoscale Research Letters* **2021**, *16*, 137.

[25] Q. Zhou, X. Luo, Y. Li, Y. Nan, H. Deng, E. Ou, W. Xu, *International Journal of Hydrogen Energy* **2020**, *45*, 433-442.

[26] S. Venkateshwaran, S. M. Senthil Kumar, *ACS Sustainable Chemistry & Engineering* **2022**, *10*, 5258-5267.

[27] S. Gratious, A. Karmakar, D. Kumar, S. Kundu, S. Chakraborty, S. Mandal, *Nanoscale* **2022**.

[28] P. Sundara Venkatesh, N. Kannan, M. Ganesh Babu, G. Paulraj, K. Jeganathan, *International Journal of Hydrogen Energy* **2022**.

[29] X. Shang, J.-Q. Chi, S.-S. Lu, B. Dong, X. Li, Y.-R. Liu, K.-L. Yan, W.-K. Gao, Y.-M. Chai, C.-G. Liu, *International Journal of Hydrogen Energy* **2017**, *42*, 4165-4173.

[30] T. P. Nguyen, S. Y. Kim, T. H. Lee, H. W. Jang, Q. V. Le, I. T. Kim, *Applied Surface Science* **2020**, *504*, 144389.

[31] H. Li, A. Li, Z. Peng, X. Fu, *Applied Surface Science* **2019**, *487*, 972-980.

[32] L. Sun, M. Gao, Z. Jing, Z. Cheng, D. Zheng, H. Xu, Q. Zhou, J. Lin, *Chemical*

Engineering Journal **2022**, *429*, 132187.

[33] J. Cho, M. Kim, H. Seok, G. H. Choi, S. S. Yoo, N. C. Sagaya Selvam, P. J. Yoo, T. Kim, *ACS Applied Materials & Interfaces* **2022**, *14*, 24008-24019.

[34] Y. Zhao, J. Li, J. Huang, L. Feng, L. Cao, Y. Feng, Z. Zhang, Y. Xie, H. Wang, *Advanced Materials Interfaces* **2020**, *7*, 2001196.

[35] W. Huang, S. Su, Y. Liu, J. Li, M. Wang, Z. Hou, X. Gao, X. Wang, R. Nötzel, G. Zhou, Z. Zhang, J. Liu, *Journal of Materials Science* **2021**, *56*, 1551-1560.

[36] R. Chen, M. Ma, Y. Luo, L. Qian, S. Wan, S. Xu, X. She, *Transactions of Tianjin University* **2022**.

[37] J. Rong, Y. Ye, J. Cao, X. Liu, H. Fan, S. Yang, M. Wei, L. Yang, J. Yang, Y. Chen, *Applied Surface Science* **2022**, *579*, 152216.

[38] aZ. Luo, J. Li, Y. Li, D. Wu, L. Zhang, X. Ren, C. He, Q. Zhang, M. Gu, X. Sun, *Advanced Energy Materials* **2022**, *12*, 2103823; bY. Li, X. Tan, H. Tan, H. Ren, S. Chen, W. Yang, S. C. Smith, C. Zhao, *Energy & Environmental Science* **2020**, *13*, 1799-1807; cJ. K. Nørskov, T. Bligaard, A. Logadottir, J. R. Kitchin, J. G. Chen, S. Pandelov, U. Stimming, *Journal of The Electrochemical Society* **2005**, *152*, J23.

[39] G. Kresse, J. Hafner, *Physical Review B* **1993**, *47*, 558-561.

[40] J. P. Perdew, K. Burke, M. Ernzerhof, *Physical Review Letters* **1996**, *77*, 3865-3868.

[41] S. L. Dudarev, G. A. Botton, S. Y. Savrasov, C. J. Humphreys, A. P. Sutton, *Physical Review B* **1998**, *57*, 1505-1509.

[42] A. Jain, S. P. Ong, G. Hautier, W. Chen, W. D. Richards, S. Dacek, S. Cholia, D. Gunter, D. Skinner, G. Ceder, K. A. Persson, *APL Materials* **2013**, *1*, 011002.

[43] M. Wang, A. Navrotsky, *Solid State Ionics* **2005**, *176*, 1181.

[44] J. W. Furness, A. D. Kaplan, J. Ning, J. P. Perdew, J. Sun, *The Journal of Physical Chemistry Letters* **2020**, *11*, 8208-8215.

[45] aA. V. Krukau, O. A. Vydrov, A. F. Izmaylov, G. E. Scuseria, *The Journal of Chemical Physics* **2006**, *125*, 224106; bB. Ward-O'Brien, P. D. McNaughter, R. Cai, A. Chattopadhyay, J. M. Flitcroft, C. T. Smith, D. J. Binks, J. M. Skelton, S. J. Haigh, D. J. Lewis, *Nano Letters* **2022**.

[46] aP. E. Blöchl, *Physical Review B* **1994**, *50*, 17953-17979; bG. Kresse, D. Joubert, *Physical Review B* **1999**, *59*, 1758-1775.

[47] H. J. Monkhorst, J. D. Pack, *Physical Review B* **1976**, *13*, 5188-5192.

[48] K. Momma, F. Izumi, *Journal of Applied Crystallography* **2011**, *44*, 1272-1276.
