# Supplementary material for: A Low‐Temperature Synthetic Route Toward a High‐Entropy 2D Hexernary Transition Metal Dichalcogenide for Hydrogen Evolution Electrocatalysis
Source: Adv Sci (Weinh). 2023 Mar 23;10(14):2204488. doi: 10.1002/advs.202204488 (PMC10190663; doi:10.1002/advs.202204488)

checkCIF/PLATON report

Structure factors have been supplied for datablock(s) m_mb-mndtc3_c2_col1__twin1_hklf4

THIS REPORT IS FOR GUIDANCE ONLY. IF USED AS PART OF A REVIEW PROCEDURE FOR PUBLICATION, IT SHOULD NOT REPLACE THE EXPERTISE OF AN EXPERIENCED CRYSTALLOGRAPHIC REFEREE.

No syntax errors found.   CIF dictionary   Interpreting this report

Datablock: m_mb-mndtc3_c2_col1__twin1_hklf4

Bond precision:	C-C = 0.0045 A		Wavelength=0.68890
Cell:	a=21.1153(4)	b=15.1249(3)	c=12.7498(2)
	alpha=90	beta=101.9419(19)	gamma=90
Temperature:	293 K		

	Calculated	Reported
Volume	3983.74(13)	3983.72(13)
Space group	C 2/c	C 1 2/c 1
Hall group	-C 2yc	-C 2yc
Moiety formula	C20 H40 Mn2 N4 O2 S8, 2(C H2 Cl2)	C20 H40 Mn2 N4 O2 S8, 2(C H2 Cl2)
Sum formula	C22 H44 Cl4 Mn2 N4 O2 S8	C22 H44 Cl4 Mn2 N4 O2 S8
Mr	904.77	904.77
Dx,g cm-3	1.509	1.509
Z	4	4
Mu (mm-1)	1.222	1.243
F000	1864.0	1864.0
F000’	1872.95	
h,k,lmax	28,20,17	28,20,17
Nref	5159	11533
Tmin,Tmax	0.769,0.856	0.469,1.000
Tmin’	0.697	

Correction method= # Reported T Limits: Tmin=0.469 Tmax=1.000
AbsCorr = MULTI-SCAN

Data completeness= 2.236   Theta(max)= 27.735

R(reflections)= 0.0541(8917)   wR2(reflections)= 0.1553(11533)

S = 1.005   Npar= 205

The following ALERTS were generated. Each ALERT has the format
test-name_ALERT_alert-type_alert-level.
Click on the hyperlinks for more details of the test.

Alert level C

```
PLAT910_ALERT_3_C Missing # of FCF Reflection(s) Below Theta(Min).          6 Note
PLAT911_ALERT_3_C Missing FCF Refl Between Thmin & STh/L=    0.600        130 Report
PLAT913_ALERT_3_C Missing # of Very Strong Reflections in FCF ....         55 Note
PLAT918_ALERT_3_C Reflection(s) with I(obs) much Smaller I(calc) .          8 Check
```

Alert level G

```
ABSMU01_ALERT_1_G  Calculation of _exptl_absorpt_correction_mu
                specified not performed for this radiation type.
PLAT002_ALERT_2_G Number of Distance or Angle Restraints on AtSite          3 Note
PLAT019_ALERT_1_G _diffrn_measured_fraction_theta_full/*_max < 1.0      0.987 Report
PLAT072_ALERT_2_G SHELXL First  Parameter in WGHT  Unusually Large       0.12 Report
PLAT092_ALERT_4_G Check: Wavelength Given is not Cu,Ga,Mo,Ag,In Ka    0.68890 Ang.
PLAT176_ALERT_4_G The CIF-Embedded .res File Contains SADI Records          1 Report
PLAT199_ALERT_1_G Reported _cell_measurement_temperature ..... (K)        293 Check
PLAT200_ALERT_1_G Reported   _diffrn_ambient_temperature ..... (K)        293 Check
PLAT232_ALERT_2_G Hirshfeld Test Diff (M-X)  Mn1      --S3       .        5.2 s.u.
PLAT302_ALERT_4_G Anion/Solvent/Minor-Residue Disorder (Resd  2  )        33% Note
PLAT860_ALERT_3_G Number of Least-Squares Restraints ............           1 Note
PLAT870_ALERT_4_G ALERTS Related to Twinning Effects Suppressed ..          ! Info
PLAT941_ALERT_3_G Average HKL Measurement Multiplicity ..........         2.3 Low
PLAT984_ALERT_1_G The Cl-f'=    0.1253 Deviates from the B&C-Value     0.1420 Check
PLAT984_ALERT_1_G The Mn-f'=    0.2891 Deviates from the B&C-Value     0.3307 Check
PLAT984_ALERT_1_G The  N-f'=    0.0037 Deviates from the B&C-Value     0.0056 Check
PLAT984_ALERT_1_G The  O-f'=    0.0071 Deviates from the B&C-Value     0.0101 Check
PLAT984_ALERT_1_G The  S-f'=    0.1048 Deviates from the B&C-Value     0.1187 Check
PLAT985_ALERT_1_G The Mn-f"=    0.6903 Deviates from the B&C-Value     0.6927 Check
```

0 **ALERT level A** = Most likely a serious problem - resolve or explain
0 **ALERT level B** = A potentially serious problem, consider carefully
4 **ALERT level C** = Check. Ensure it is not caused by an omission or oversight
19 **ALERT level G** = General information/check it is not something unexpected

10 ALERT type 1 CIF construction/syntax error, inconsistent or missing data
3 ALERT type 2 Indicator that the structure model may be wrong or deficient
6 ALERT type 3 Indicator that the structure quality may be low
4 ALERT type 4 Improvement, methodology, query or suggestion
0 ALERT type 5 Informative message, check

It is advisable to attempt to resolve as many as possible of the alerts in all categories. Often the minor alerts point to easily fixed oversights, errors and omissions in your CIF or refinement strategy, so attention to these fine details can be worthwhile. In order to resolve some of the more serious problems it may be necessary to carry out additional measurements or structure refinements. However, the purpose of your study may justify the reported deviations and the more serious of these should normally be commented upon in the discussion or experimental section of a paper or in the "special_details" fields of the CIF. checkCIF was carefully designed to identify outliers and unusual parameters, but every test has its limitations and alerts that are not important in a particular case may appear. Conversely, the absence of alerts does not guarantee there are no aspects of the results needing attention. It is up to the individual to critically assess their own results and, if necessary, seek expert advice.

Publication of your CIF in IUCr journals

A basic structural check has been run on your CIF. These basic checks will be run on all CIFs submitted for publication in IUCr journals (*Acta Crystallographica*, *Journal of Applied Crystallography*, *Journal of Synchrotron Radiation*); however, if you intend to submit to *Acta Crystallographica Section C* or *E* or *IUCrData*, you should make sure that full publication checks are run on the final version of your CIF prior to submission.

Publication of your CIF in other journals

Please refer to the *Notes for Authors* of the relevant journal for any special instructions relating to CIF submission.

PLATON version of 28/11/2022; check.def file version of 28/11/2022

Datablock m_mb-mndtc3_c2_col1__twin1_hklf4 - ellipsoid plot

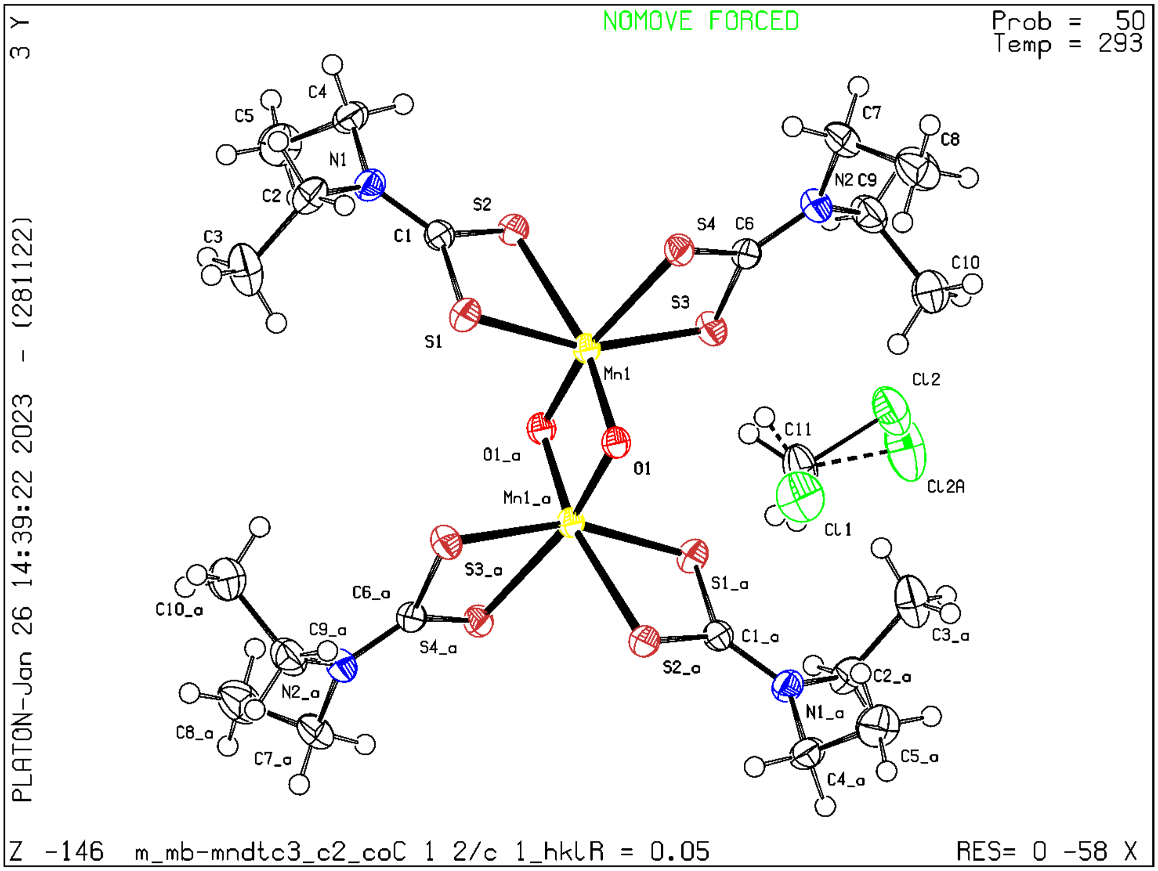

Supplement: Supplementary file 3 — Supporting Information [file ADVS-10-2204488-s001.pdf]
